# Triplet exciton recycling in organic solar cells enabled by an extrinsic triplet–triplet annihilation dopant

**DOI:** 10.1039/d6el00075d

**Published:** 2026-10-12

**Authors:** Darcy M. L. Unson, Rachel C. Kilbride, Jeannine Grüne, Daniel T. W. Toolan, Pascal Kaienburg, Subhrangsu Mukherjee, Sangcheol Yoon, Nakul Jain, Hanbo Yang, Jarvist M. Frost, Harald Ade, Thuc-Quyen Nguyen, Andreas Sperlich, Vladimir Dyakonov, Neil C. Greenham, Alexander J. Gillett

**Affiliations:** a Cavendish Laboratory, University of Cambridge J J Thomson Avenue Cambridge CB3 0HE UK; b School of Mathematics and Physical Sciences, University of Sheffield Brook Hill Sheffield S3 7HF UK; c Department of Physics, The University of Warwick Coventry CV4 7AL UK; d XMaS, The UK X-ray Materials Science Facility, European Synchrotron Radiation Facility F-38043 Grenoble France; e Experimental Physics 6, Julius Maximilian University of Würzburg Am Hubland Würzburg 97074 Germany; f Department of Materials, University of Manchester Booth St East Manchester M13 9SS UK; g Clarendon Laboratory, Department of Physics, University of Oxford Parks Road OX1 3PU Oxford UK; h Department of Physics and Organic and Carbon Electronics Laboratories (ORaCEL), North Carolina State University Raleigh NC 27695 USA; i Center for Polymers and Organic Solids, Department of Chemistry and Biochemistry, University of California at Santa Barbara Santa Barbara CA 93106 USA; j Department of Physics, Chemistry and Biology (IFM), Linköping University 581 83 Linköping Sweden alexander.gillett@liu.se; k Department of Physics, Imperial College London London SW7 2BW UK

## Abstract

Enabled by the optimisation of non-fullerene electron acceptors (NFAs), organic solar cells (OSCs) now exhibit power conversion efficiencies exceeding 20%. However, to further increase OSC performance, it is essential to target the areas of device function where significant losses remain. One key factor now limiting OSCs is charge recombination *via* the spin-triplet exciton state on the NFA component. Here, we show that by doping the archetypal triplet–triplet annihilation (TTA) material rubrene into a model PM6:Y6 blend, the triplet excitons formed on Y6 *via* non-geminate charge recombination can be transferred to rubrene. The rubrene triplets then undergo TTA to form singlet excitons followed by recycling into free charge carriers. We further reveal a complex evolution in the bulk heterojunction morphology upon the addition of rubrene, including the formation of mixed domains containing all three materials. We propose that the presence of rubrene in this mixed region near the PM6:Y6 interface enables it to rapidly accept the triplets generated on Y6. Our work therefore provides a proof of concept for triplet recycling by an extrinsic TTA dopant in OSCs, which can inspire further innovative strategies for triplet exciton management in both OSCs and photon upconversion systems.

## Abstract 2

By doping the triplet–triplet annihilation material rubrene into a PM6:Y6 organic solar cell, the triplet excitons formed *via* non-geminate charge recombination can be recycled.

## Graphical abstract

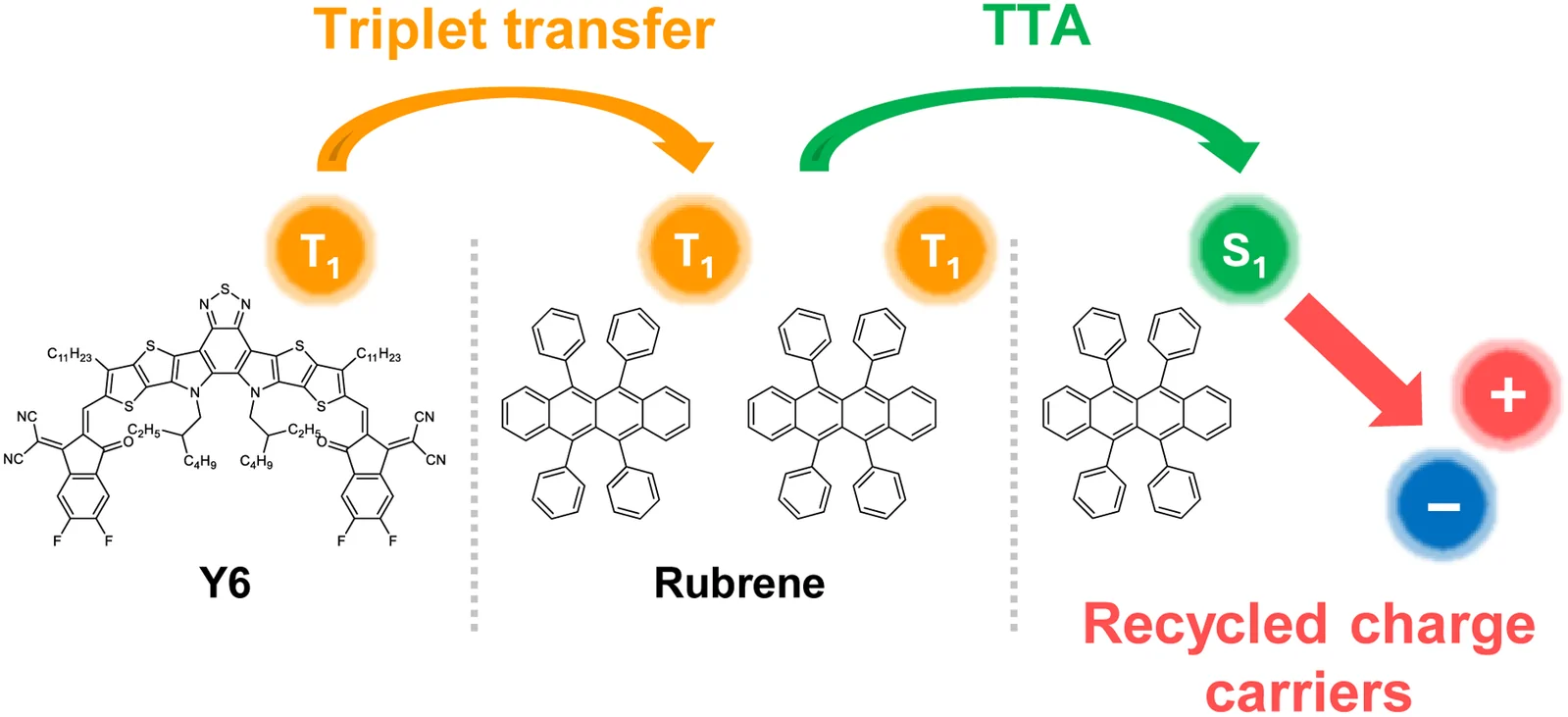

**Broader context**

This work addresses a fundamental loss pathway that limits the performance of state-of-the-art organic solar cells (OSCs): non-radiative recombination *via* spin-triplet excitons. While recent advances in non-fullerene acceptors have enabled power conversion efficiencies exceeding 20%, further progress is increasingly constrained by these intrinsic loss channels. Here, we introduce and experimentally validate a new strategy for mitigating such losses using an extrinsic triplet–triplet annihilation (TTA) dopant. By incorporating rubrene into a model PM6:Y6 bulk heterojunction, we demonstrate as a proof-of-concept that triplet excitons formed *via* charge recombination can be harvested, transferred, and upconverted into singlet states, which are subsequently recycled into free charge carriers. This represents a conceptual shift from attempting to suppress triplet formation toward actively managing and repurposing these states. Beyond photovoltaics, these insights have important implications for the design of photon upconversion and other optoelectronic systems where triplet management is critical.

## 1. Introduction

Since the widespread adoption of non-fullerene electron acceptor (NFA) materials, the power conversion efficiency (PCE) of sunlight into electricity for organic solar cells (OSCs) has rapidly increased and now exceeds 20%.^1–3^ However, the performance of OSCs is still lower than inorganic photovoltaic technologies, such as crystalline silicon and perovskites, where PCEs exceed 25%.^4^ The key metric requiring improvement in OSCs is the low open circuit voltage (*V*_OC_) relative to their optical bandgap, which can be attributed to the dominance of non-radiative charge recombination processes in the donor–acceptor bulk heterojunction (BHJ).^5–7^ Thus, to further advance OSC performance, new strategies are required to minimise non-radiative recombination and improve the luminescence yields of OSC materials and devices.

The poor luminescence yields of OSCs can be attributed to two key factors. The first is the low intrinsic photoluminescence quantum efficiency (PLQE) of the low-band-gap component in the BHJ blend, typically the NFA, which sets the upper limit for the maximum device luminescence yield that can be achieved.^5,6^ As the most efficient OSCs use narrow-band-gap NFA materials with photoluminescence peaks beyond 900 nm,^1–3^ these systems are intrinsically limited by the energy gap law,^8,9^ which predicts that the non-radiative rate increases rapidly as the emission peak shifts to wavelengths longer than 700 nm.^10^ Thus, for narrow-band-gap NFAs, which generally have PLQEs of a few percent in a pristine thin film,^5^ there is limited scope for the significant improvements required in the PLQE, likely to several tens of percent, for a noticeable boost in the *V*_OC_.

The second contributor to the low luminescence yields in OSCs is the recombination of free charge carriers to spin-triplet excitons (T_1_).^11^ In the simple spin-statistical approximation originally proposed by the organic light emitting diode (OLED) field,^12^ 75% of the recombination events involving spin-uncorrelated electrons and holes will form an excitation with spin-triplet character, with only 25% forming a state with spin-singlet character. Indeed, there have been many reports of significant recombination to the T_1_ state in a wide range of OSC systems,^13–19^ with several even suggesting that the fraction can exceed 75%.^11,20,21^ As triplet states generally decay without photon emission,^12^ it has been estimated that recombination *via* T_1_ (typically on the NFA component) can reduce the *V*_OC_ by up to 60 mV.^11^ Therefore, to reduce the non-radiative voltage losses in OSCs and improve the *V*_OC_, it is essential to block or mitigate charge recombination to T_1_.

The T_1_ state located on the NFA is often the lowest-energy intramolecular excitation in the OSC system.^11^ This typically places it below the charge-transfer (CT) states, meaning it is thermodynamically favourable for charge recombination to ultimately proceed *via* T_1_. Thus, it is extremely difficult to completely block the population of T_1_. Therefore, it is necessary to develop new strategies to mitigate the negative effects of T_1_ population after this state is formed. One promising strategy involves utilising triplet–triplet annihilation (TTA) to recycle two T_1_ states back into one singlet exciton (S_1_) state, which can either emit a photon or undergo charge transfer to re-form free charge carriers in the BHJ. Y6 derivatives, such as the material Y11, have shown extremely high TTA rate constants (>10^−10^ cm^3^ s^−1^), which results in detectable nanosecond delayed fluorescence from TTA in a pristine Y11 film.^20^ However, the monomolecular T_1_ lifetime of Y6-type materials in thin films was found to be extremely short: on the order of tens to hundreds of nanoseconds. Kinetic modelling showed that under 1-sun excitation densities, fast monomolecular T_1_ decay to the ground state would outcompete TTA due to the low T_1_ concentration in the film.^20^ Thus, for the current range of ‘Y-series’ NFAs, this ‘intrinsic’ TTA behaviour is unlikely to provide much benefit to OSC device performance under realistic 1-sun operating conditions.

In this work, we show that rather than relying on the intrinsic TTA of the NFA to facilitate triplet recycling, a known TTA mediator with a suitably long T_1_ lifetime, here the archetypal TTA material rubrene^22^ (T_1_ lifetime = 750 µs),^23^ can instead be added into the model PM6:Y6 BHJ blend as an ‘extrinsic’ TTA dopant (chemical structures in Fig. 1a). Using photoluminescence-detected magnetic resonance (PLDMR) studies, we show that the T_1_ state on rubrene directly mediates the near-infrared (NIR) luminescence of the PM6:Y6 BHJ blend by recycling T_1_ states. The presence of triplet recycling in the PM6:Y6:rubrene ternary blend is further supported by transient absorption (TA) spectroscopy, which provides detailed information about the underlying mechanism primarily involving the transfer of T_1_ generated on Y6 to rubrene. Through a comprehensive microstructure characterisation of the PM6:Y6:rubrene film, we investigate the underlying morphological features that enable the triplet recycling to take place. We reveal that rubrene does not phase separate to form large crystalline rubrene aggregates but is instead located within mixed PM6:Y6:rubrene domains; though it is not possible to determine the precise form of the rubrene molecules in these mixed domains, our spectroscopic results suggest that it is sufficient to enable the Dexter transfer of T_1_ from Y6 to rubrene, followed by TTA. Finally, we analyse how the addition of rubrene impacts the OSC device performance. Thus, our findings provide a proof-of-concept demonstration of how a TTA material can be doped into an OSC blend to mediate against losses *via* T_1_ states. This has great relevance not only for OSC devices, but also for understanding the emerging class of photon upconversion systems that make use of a BHJ blend for triplet sensitisation of a TTA material.^24,25^

**Fig. 1:**
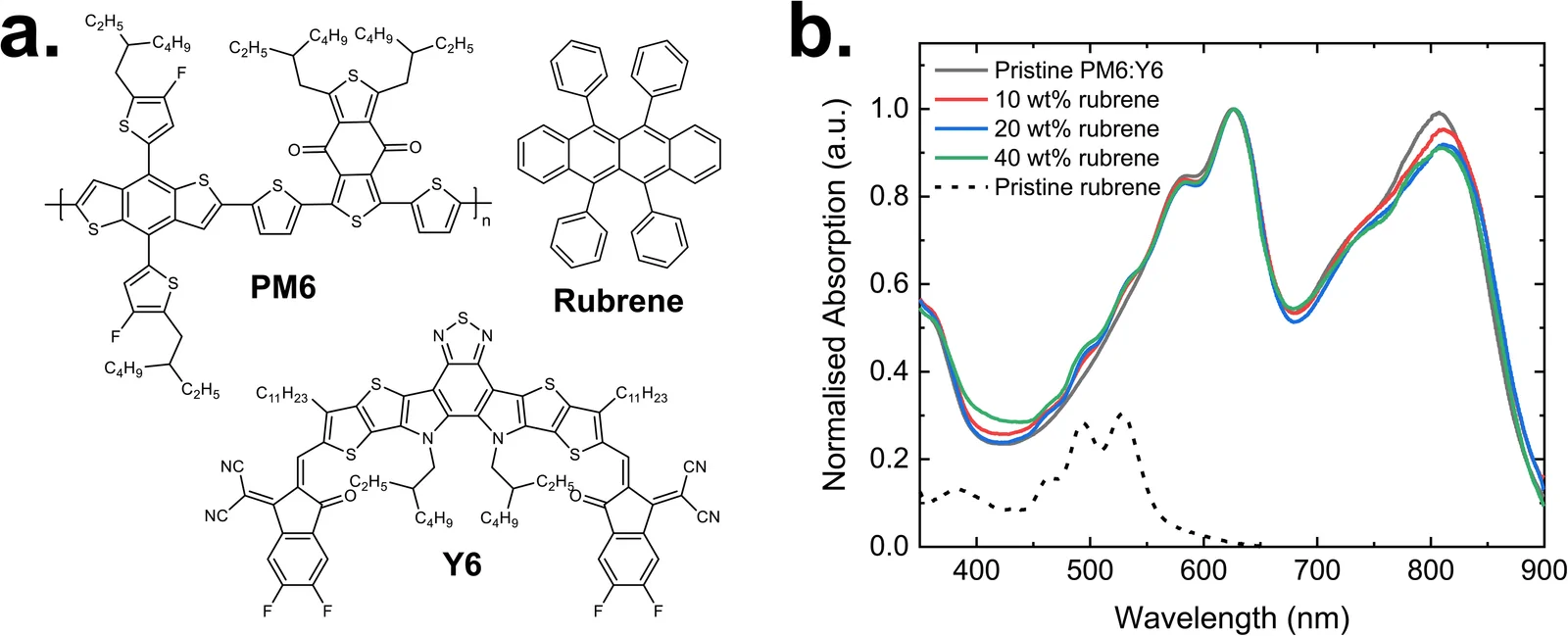
(a) The chemical structures of the materials used in this work: PM6, Y6, and rubrene. (b) The normalised steady-state absorption spectrum of the pristine PM6:Y6 and rubrene films, as well as PM6:Y6 films doped with 10, 20, and 40 wt% rubrene. The blend absorption spectra have been normalised to the peak of the PM6 absorption.

## 2. Results and discussion

### 2.1 Steady-state characterisation of thin films

Owing to the complex nature of an OSC device doped with the TTA material rubrene, we first discuss the photophysical and morphological properties of the BHJ layer alone. To understand the behaviour of rubrene when combined with the electron donor and acceptor materials commonly used in OSCs, we have fabricated ternary BHJ blend films comprising PM6, Y6, and rubrene. To explore the impact of rubrene concentration on the TTA upconversion properties of the BHJ film, we have also used three different rubrene concentrations in the PM6:Y6:rubrene samples. These comprise 10, 20, and 40 wt% rubrene, as well as a control film with 0 wt% rubrene, which for clarity we will refer to as the ‘pristine’ PM6:Y6 sample hereafter.

Since rubrene possesses a much lower peak absorption coefficient (∼2 × 10^4^ cm^−1^)^26^ than PM6 and Y6 (∼1.1 × 10^5^ cm^−1^),^27^ and has a wider band gap, the addition of significant quantities of rubrene into the PM6:Y6 blend would be expected to greatly reduce the light harvesting abilities of the OSC device for a constant film thickness. Thus, as strong light absorption is necessary for efficient photocurrent generation in an OSC, we have chosen to retain the same absolute PM6 : Y6 (1 : 1.2 blend ratio) concentration of 15.4 mg mL^−1^ in the precursor solutions used to make the pristine and rubrene-doped BHJ films. For the PM6:Y6:rubrene films, we have added additional rubrene to the PM6:Y6 solution to obtain the desired doping wt%; rubrene loadings are expressed as a percentage of the combined mass of PM6 and Y6, excluding rubrene. For example, the precursor solution for the 20 wt% rubrene film contained 15.4 mg per mL PM6:Y6 and 3.08 mg per mL rubrene, giving a total solution concentration of 18.48 mg mL^−1^. This has the desired effect of maintaining a similar absolute optical density for the pristine PM6:Y6 and PM6:Y6:rubrene films (Fig. S1). However, as the total precursor solution concentration is raised, the film thickness consequently increases from 170 nm in pristine PM6:Y6, to 200, 218, and 233 nm in the 10, 20, and 40 wt% rubrene films, respectively.

Fig. 1b shows the steady-state absorption spectrum of the PM6:Y6:rubrene films, as well as the reference pristine PM6:Y6 and evaporated pristine rubrene films. The spectra of samples containing PM6:Y6 have been normalised to the peak of the PM6 absorption band. Upon the addition of rubrene, three new vibronic bands from rubrene between 450 and 550 nm become weakly visible on top of the PM6 and Y6 absorption features. However, owing to the low absorption coefficient of rubrene,^26^ it does not provide a significant enhancement to the light absorption of the blend film in this spectral region. Whilst there is no noticeable change in the PM6 absorption band upon the addition of rubrene, we observe some changes in the Y6 absorption features around 800 nm. Here, there is a slight decrease in the relative intensity of the Y6 absorption compared to PM6 and a subtle change in the band shape. As the spectral shape and position of the absorption band in Y6 are known to be extremely sensitive to the local molecular packing and morphology,^28^ these changes provide a preliminary suggestion that the addition of rubrene has some impact on the microstructure of Y6 and that close intermolecular interactions may be present between Y6 and rubrene in the BHJ.

To check for evidence of TTA upconversion in the PM6:Y6:rubrene blend, we performed steady-state photoluminescence (PL) measurements on the BHJ film with selective excitation of Y6 at 800 nm (Fig. S2a). Curiously, we do not detect any rubrene PL at 570 nm from the 10, 20, or 40 wt% rubrene samples, though we find upconversion emission in a Y6:rubrene bilayer (Fig. S2b), confirming that the TTA upconversion process should be possible in this material combination. This contrasts with a recent report that used a BHJ blend consisting of a PBQx-TCl polymer electron donor and PYIT1 polymerised electron acceptor to sensitise the rubrene triplet state for highly efficient solid-state TTA photon upconversion.^24^ Based on our observation of no upconverted rubrene PL in the PM6:Y6:rubrene blends, we conclude that either there is no triplet recycling by rubrene in the system, or a process other than fluorescence is quenching the rubrene S_1_ states formed after TTA.

In the BHJ-sensitised rubrene upconversion system mentioned above, we note that the PBQx-TCl electron donor possesses a wider band gap than the PM6 material used in our work. Thus, whilst there is little spectral overlap of the rubrene PL with the PBQx-TCl absorption band,^24^ we find that the rubrene PL overlaps near-perfectly with the PM6 absorption (Fig. S3). This provides the possibility for efficient Förster resonance energy transfer (FRET) from the rubrene S_1_ states formed *via* TTA to PM6, which is not present with PBQx-TCl. Whilst it should be energetically possible for rubrene to transfer electrons to PM6 and Y6 (Fig. S4), we cannot selectively photogenerate excitons on rubrene to probe this process due to the overlapping, and much stronger, absorption bands of PM6 and Y6. However, our TA studies on the Y6:rubrene (Fig. S5) and PM6:rubrene (Fig. S6) BHJ blends find evidence for hole transfer from Y6 to rubrene, but no clear indication of hole transfer from PM6 to rubrene.

To explore the FRET hypothesis further, we have calculated the Förster radius (*R*_0_) and rate constant (*k*_FRET_) for the rubrene:PM6 combination (calculation details in the SI). Owing to the significant overlap between the rubrene emission and PM6 absorption, we obtain a large *R*_0_ of 5.8 nm, with a high *k*_FRET_ of 1.73 × 10^12^ s^−1^. Thus, it is plausible that we observe no upconverted PL in our PM6:Y6:rubrene system due to the rapid quenching of any rubrene S_1_ states by FRET to PM6, followed by electron transfer to Y6 to regenerate free electrons and holes. Nevertheless, electron transfer from rubrene to PM6 and/or Y6 may also be possible, and testing this hypothesis necessitates a more detailed photophysical study to ascertain whether triplet recycling is occurring in the PM6:Y6:rubrene BHJ.

### 2.2 Photoluminescence-detected magnetic resonance

As there is no detectable rubrene PL in the PM6:Y6:rubrene BHJ blends, it is not trivial to demonstrate that triplet recycling by rubrene is occurring in the system. However, a powerful technique that can reveal how paramagnetic electronic excitations (*i.e.* T_1_ states, spin-triplet CT (^3^CT) states, and free polarons), are contributing to the luminescence of a sample is PLDMR.^16,17,29,30^ In PLDMR, a sample is placed within an external magnetic field and irradiated with microwaves (here, X-band) of a fixed frequency. The magnetic field energetically splits the triplet state into the three spin sublevels T_+1_, T_0_, and T_−1_ through the Zeeman interaction and is swept over a certain field range. Once the sublevel splitting is resonant with the microwave photon energy, a spin transition is induced which alters the relative population of the triplet sublevels. As each triplet sublevel couples differently to the bright spin-singlet states in the system, for example *via* sublevel-dependent TTA, the PL yield of the sample is changed by the resonant microwaves.^31^ This provides information about which high spin states are acting as precursors to the emission process in a sample.

First, we measured and simulated the PLDMR spectrum of pristine PM6, Y6, and rubrene films to detect the spectral signatures of their high spin excited states (Fig. 2 and S7–S9). We note that the zero-field-splitting (ZFS) *D*-parameter of the T_1_ state (Table S2), which is related to the delocalisation between the spins, is different for Y6 (950 MHz) and rubrene (1550 MHz). The larger *D*-parameter for rubrene indicates the presence of a more localised T_1_ state and results in a broader spectral width compared to Y6 for the full field (270–410 mT) T_1_ spectrum. Importantly, the different *D*-parameter value for Y6 and rubrene means that their half field (160–172 mT) signals, resulting from the formally spin-forbidden Δ*m*_s_ = ±2 transition, are located at different field positions: 167.5 mT for Y6 and 165 mT for rubrene. This provides an effective way to distinguish between the contribution of the Y6 and rubrene triplets to the PL of the PM6:Y6:rubrene BHJ sample.

**Fig. 2:**
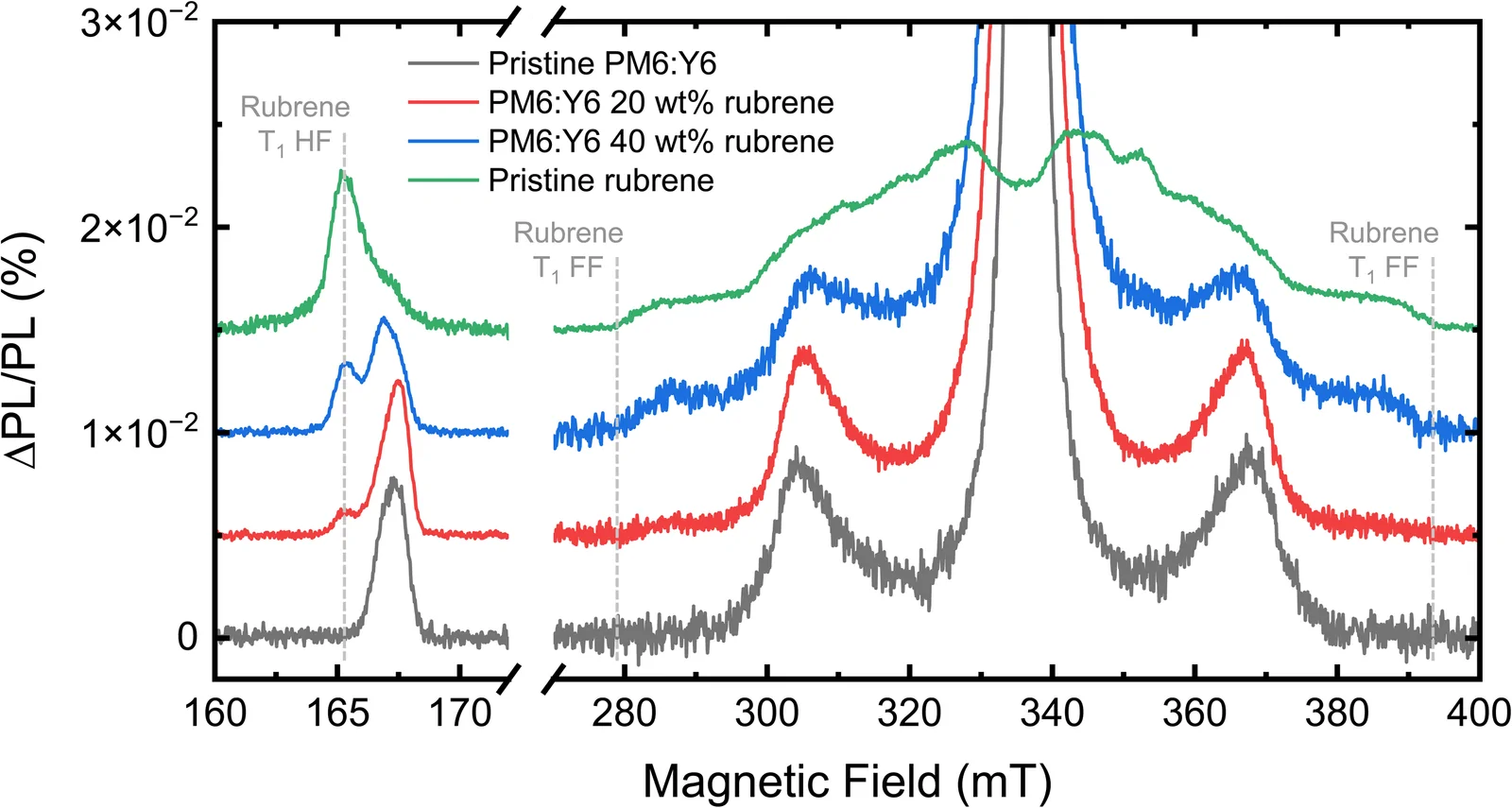
The PLDMR spectra of a pristine PM6:Y6 film, PM6:Y6 films doped with 20 and 40 wt% rubrene, and a pristine rubrene film, offset for clarity. The dashed grey lines at 165, 279, and 393 mT are guides-to-the-eye to highlight the positions of the rubrene half-field (HF) and full-field (FF) signals. To show the spectral features more clearly, the FF spectra for pristine PM6:Y6 and PM6:Y6 with 20 wt% rubrene have been enlarged by a factor of 3, whilst PM6:Y6 with 40 wt% rubrene has been enlarged by a factor of 4. The rubrene FF spectrum has been scaled by a factor of 0.3. The HF spectra are unscaled for all samples. The pristine PM6:Y6, and PM6:Y6 with 20 wt% and 40 wt% rubrene samples were measured using continuous wave laser excitation at 785 nm, with a power of 50 mW, and detection at >830 nm. The pristine rubrene film was measured using continuous wave laser excitation at 532 nm, with a power of 80 mW, and detection at >561 nm. All measurements were carried out at 10 K.

Turning to the BHJ blend films, we measured the reference pristine PM6:Y6 film with CW excitation at 532 nm, for preferential pumping of PM6, and 785 nm, for selective excitation of Y6 (Fig. 2 and S10). We record an identical spectrum in each case, confirming that the excitation wavelength does not have a significant influence on the PLDMR measurement for this sample. The spectrum we obtained for pristine PM6:Y6 is in good agreement with our previous report;^30^ we observe a strong contribution from ^3^CT states/polarons through the narrow and intense central peak at 336 mT, as well as the presence of T_1_ states only located on Y6, as seen by the distinct half- (167.5 mT) and full- (304 and 368 mT) field transitions of Y6. Moving to the rubrene-doped BHJ blends, in Fig. 2 we also present the PLDMR spectra for the 20 and 40 wt% PM6:Y6:rubrene films. Importantly, we performed these measurements with 785 nm excitation and an 830 nm long-pass filter in front of the detector. This ensures that only Y6 is excited, with the luminescence only detected from the Y6 S_1_ or spin-singlet CT (^1^CT) states in the blend, as neither rubrene nor PM6 display emission in this region (Fig. S3).

In the 20 wt% PM6:Y6:rubrene film, we observe the same spectral features as pristine PM6:Y6, namely the presence of ^3^CT states/polarons and T_1_ states on Y6. However, we also notice additional signals in the 20 wt% sample: the presence of weak ‘wings’ in the full-field spectrum, extending beyond the Y6 T_1_ response to 279 and 393 mT, as well as a half-field signal at 165 mT. These new spectral features match those of the pristine rubrene film (see dashed grey lines overlaid on the spectra), confirming that the rubrene T_1_ state is both populated and linked to the NIR emission of the PM6:Y6:rubrene BHJ film. Upon the addition of 40 wt% rubrene to the blend, we find a further enhancement in the intensity of the rubrene half- and full-field signals relative to the Y6 features, suggesting that a higher rubrene doping concentration results in a greater involvement of the rubrene T_1_ in the NIR luminescence of the BHJ blend. Curiously, with the addition of 40 wt% rubrene, we note that the spectral wings of the Y6 T_1_ at 304 and 368 mT appear less sharply pronounced. Simulations of the PLDMR spectra (Fig. S11 and Table S3) reveal that this is due to a decrease in the molecular ordering parameter (*λ*_*Θ*_) of Y6. As all the PLDMR measurements were performed with the *z*-axis (out-of-plane direction) of the film parallel to the magnetic field direction (Fig. S12), the reduced ordering parameter indicates that the addition of large quantities of rubrene disrupts the π–π stacking and thus decreases the ordering of Y6 in the out-of-plane direction relative to the substrate. This further supports the notion that there are intermolecular interactions between Y6 and rubrene molecules in the BHJ.

In the PLDMR measurements, we emphasise that the experimental conditions ensure that there is no direct photoexcitation of rubrene in the PM6:Y6:rubrene film (a pristine rubrene film excited at 785 nm also showed no PLDMR response; Fig. S8), nor the detection of any rubrene PL. Thus, to explain the contribution of the rubrene T_1_ state to the NIR PL of the blend, we propose that it is populated following the non-geminate recombination of charge carriers in the BHJ. This could plausibly be *via* either Dexter transfer from T_1_ states initially generated on Y6, or direct back charge transfer from ^3^CT states in the blend (Fig. 3); we will return to this important mechanistic detail in the next section. Nonetheless, once formed, the rubrene T_1_ states can undergo TTA to generate rubrene S_1_ states. Whilst direct emission from an S_1_ state in the blend is possible, either *via* rubrene, or PM6 (or Y6) following FRET from rubrene, we consider that most S_1_ states likely re-form free charge carriers owing to the efficient charge generation in PM6:Y6. After several of the cycles shown in Fig. 3,^20^ these charges can then eventually recombine and contribute to the blend PL beyond 830 nm *via* emission from the Y6 S_1_ or ^1^CT states. Thus, the PLDMR experiment confirms that the rubrene T_1_ state is linked to the NIR luminescence of the PM6:Y6:rubrene blend.

**Fig. 3:**
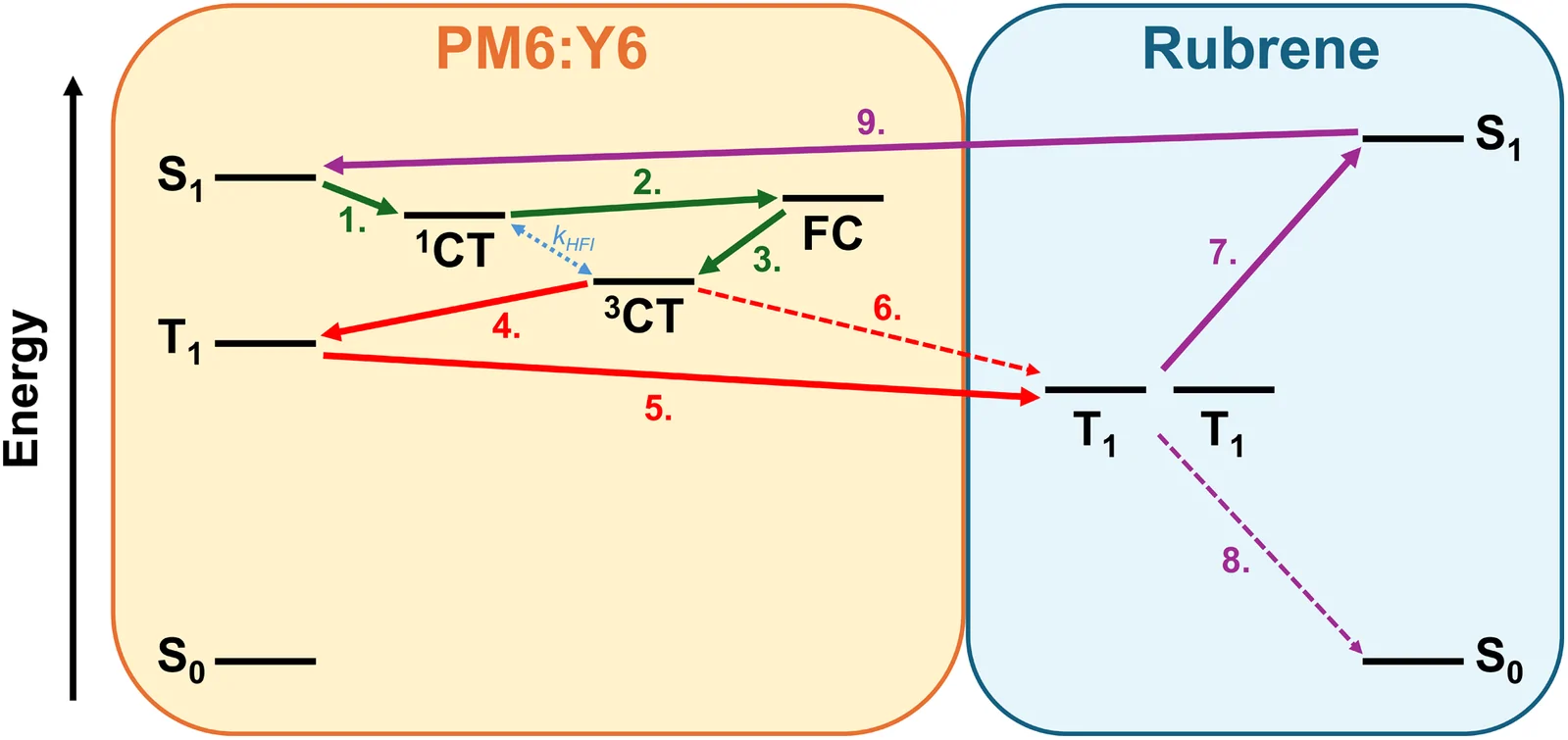
A schematic showing the proposed triplet recycling mechanism in the PM6:Y6:rubrene blend. (1) Photogenerated singlet excitons (S_1_) on PM6 and Y6 undergo charge transfer to form spin-singlet charge transfer states (^1^CT). (2) The ^1^CT states separate into free charges (FC). (3) The FCs recombine to form spin-triplet charge transfer states (^3^CT). (4) Back charge transfer takes place from the ^3^CT states to the triplet exciton (T_1_) state of Y6. (5) The Y6 T_1_ states undergo Dexter transfer to populate the rubrene T_1_. (6) As a minor pathway, direct back charge transfer from the ^3^CT states to the rubrene T_1_ is also energetically possible. (7) The rubrene T_1_ states undergo TTA to form rubrene S_1_ states. (8) Half of the rubrene T_1_ states are deactivated in the TTA process. (9) Rubrene S_1_ states transfer *via* FRET to the PM6 S_1_, which can then re-enter the cycle *via* charge transfer to ^1^CT. The potential for population transfer between the ^1^CT and ^3^CT states *via* the hyperfine interaction (HFI), which could impact the branching ratio between the singlet and triplet channels, is indicated by the blue dotted line.

### 2.3 Transient absorption spectroscopy

Whilst PLDMR provides strong evidence for the ability of rubrene to recycle triplets in the PM6:Y6:rubrene BHJ blend *via* TTA, it is not possible to discern the precise pathway and timescales of the underlying mechanism. Therefore, to better understand the photophysical processes underpinning the triplet recycling, we have conducted TA studies on the PM6:Y6:rubrene system. To begin, we address the question of whether rubrene is accepting T_1_ states *via* a Dexter-type process from Y6, or if the ^3^CT states in the blend are undergoing back charge transfer directly to the rubrene T_1_, in preference to the Y6 T_1_ state (Fig. 3).

To explore whether the T_1_ state on Y6 can transfer to rubrene, we performed triplet quenching studies on Y6 in the presence of rubrene. We note that Y6 has been reported to possess an intersystem crossing (ISC) quantum yield of ∼5%.^20^ Thus, it is possible to generate a significant population of T_1_ states on Y6 following photoexcitation, without the need for the added complication of a triplet sensitiser. However, since the Y6:rubrene BHJ film shows a strong quenching of Y6 excited states by hole transfer which reduces the ISC yield (Fig. S5b), we instead work with a Y6:rubrene bilayer film which exhibits less efficient hole transfer.^32^ To check for evidence of triplet quenching we performed nanosecond TA on the Y6:rubrene bilayer (Fig. S13). At 1–2 ns after selective excitation of Y6 at 800 nm, the only excited state remaining on Y6 is the T_1_, with a characteristic photo-induced absorption (PIA) feature centred at 1450 nm.^11^ In the presence of rubrene, we notice a clear shortening of the Y6 T_1_ lifetime, indicative of triplet quenching by rubrene. Thus, we conclude that the Y6 T_1_ state must be slightly higher in energy than the rubrene T_1_, allowing it to be quenched by Dexter transfer. Our finding is consistent with a recent report which found the T_1_ energy of several Y6 derivatives to be ∼1.2 eV using time-resolved two-photon photoemission spectroscopy,^33^ which lies above the rubrene T_1_, reported at 1.14 ± 0.02 eV.^34^ A rubrene T_1_ energy in this range is confirmed by the direct S_0_ → T_1_ absorption band measured in the presence of lanthanide nanocrystals.^35^

To investigate the feasibility of back charge transfer from ^3^CT states to the rubrene T_1_, we first note that the CT state formed between Y6 and rubrene, which is estimated to be between 1.21 and 1.28 eV,^36^ lies above the rubrene T_1_. Given that PM6 has a smaller energetic offset with rubrene and a wider band gap than Y6 (Fig. S4), any CT state formed between PM6 and rubrene is also expected to lie significantly higher than the Y6:rubrene CT state. As they comprise the largest weight fraction of the ternary system, we consider that the dominant CT state will likely be that formed between PM6 and Y6. However, the energetic offset in PM6:Y6,^37^ and thus the CT state energy, is widely debated in the OSC field, with CT energies ranging between 1.09 and 1.41 eV reported.^38–40^ Nonetheless, as our Y6:rubrene triplet quenching measurements (Fig. S13) suggest that the Y6 T_1_ state must lie above the rubrene T_1_, and a previous report has shown that back charge transfer from the PM6:Y6 ^3^CT state to the Y6 T_1_ occurs with a very high yield,^11^ we deduce that the likely energetic ordering of the states is: ^3^CT (between any two components) > Y6 T_1_ > rubrene T_1_. Thus, it appears plausible that back charge transfer from ^3^CT to the rubrene T_1_ should be energetically downhill and therefore possible.

Having confirmed that the rubrene T_1_ state can plausibly be populated *via* both Dexter transfer from the Y6 T_1_ state and back charge transfer from any ^3^CT state in the ternary system, we next examine the TA of the PM6:Y6:rubrene blend. We begin by searching for the rubrene T_1_ PIA, which is reported to have a peak around 490–510 nm,^41–43^ in the PM6:Y6 blend with 20 wt% rubrene. In the 470–530 nm spectral region, we observe a complex TA signal with several overlapping features (Fig. S14), which we discuss in detail in the SI. After a few hundred picoseconds, we note the formation of a long-lived PIA band at around 490 nm, which shows some additional nanosecond growth at around 510 nm. However, the PIA at 490 nm is weak and overlaps with other spectral features in the sample, such as the PM6 ground-state bleach (GSB). Indeed, rubrene is known to give a particularly weak TA signal, with the rubrene GSB also noticeably absent in its TA spectrum.^41,42^ Thus, whilst the presence of a PIA around 490 nm and the timescales of its formation (see below) appear to be consistent with the population of the rubrene T_1_ state, it is not possible to reliably extract information from this spectral region due to the small PIA signal size and overlap with the stronger signatures of PM6 and Y6. Therefore, to understand the triplet dynamics in the PM6:Y6:rubrene system, we instead track the behaviour of the more clearly resolved Y6 T_1_ PIA.

In Fig. 4a, we present the TA spectrum of the IR region (1250–1600 nm) for the reference pristine PM6:Y6 film with selective excitation of Y6 at 800 nm. The 10, 20, and 40 wt% PM6:Y6:rubrene films exhibit the same spectral features and are shown in Fig. S16. Consistent with a previous report, at 0.2–0.3 ps, we observe the PIA signature of hole polarons on Y6 at 1550 nm.^28^ The hole PIA is related to the fact that neat Y6 forms a significant population of intermolecular CT states immediately after photoexcitation (sometimes referred to as ‘intra-moiety excited states’, i-EX),^28,44,45^ and these CT states mediate hole transfer to PM6. As hole transfer progresses on picosecond timescales, the PIA at 1550 nm is quenched and the Y6 T_1_ PIA at 1450 nm grows in over hundreds of picoseconds. We have extracted kinetics from the spectral region associated with the Y6 T_1_ PIA (1425–1475 nm) for the pristine PM6:Y6 film (Fig. 4b). The initial decay in the first ∼100 ps is related to the quenching of the Y6 hole polaron PIA, which spectrally overlaps with the Y6 T_1_ PIA. After the Y6 hole polaron PIA has decayed, we observe the delayed growth of the Y6 T_1_ PIA. A fluence series confirms the signature of non-geminate charge recombination to the Y6 T_1_ in pristine PM6:Y6, where the formation of T_1_ states becomes faster and more prominent as the excitation fluence is raised.^11^ The formation of Y6 T_1_ states is followed by their quenching *via* triplet-charge annihilation (TCA) after ∼1 ns, which again becomes more noticeable at higher fluences due to the higher charge density in the blend.

**Fig. 4:**
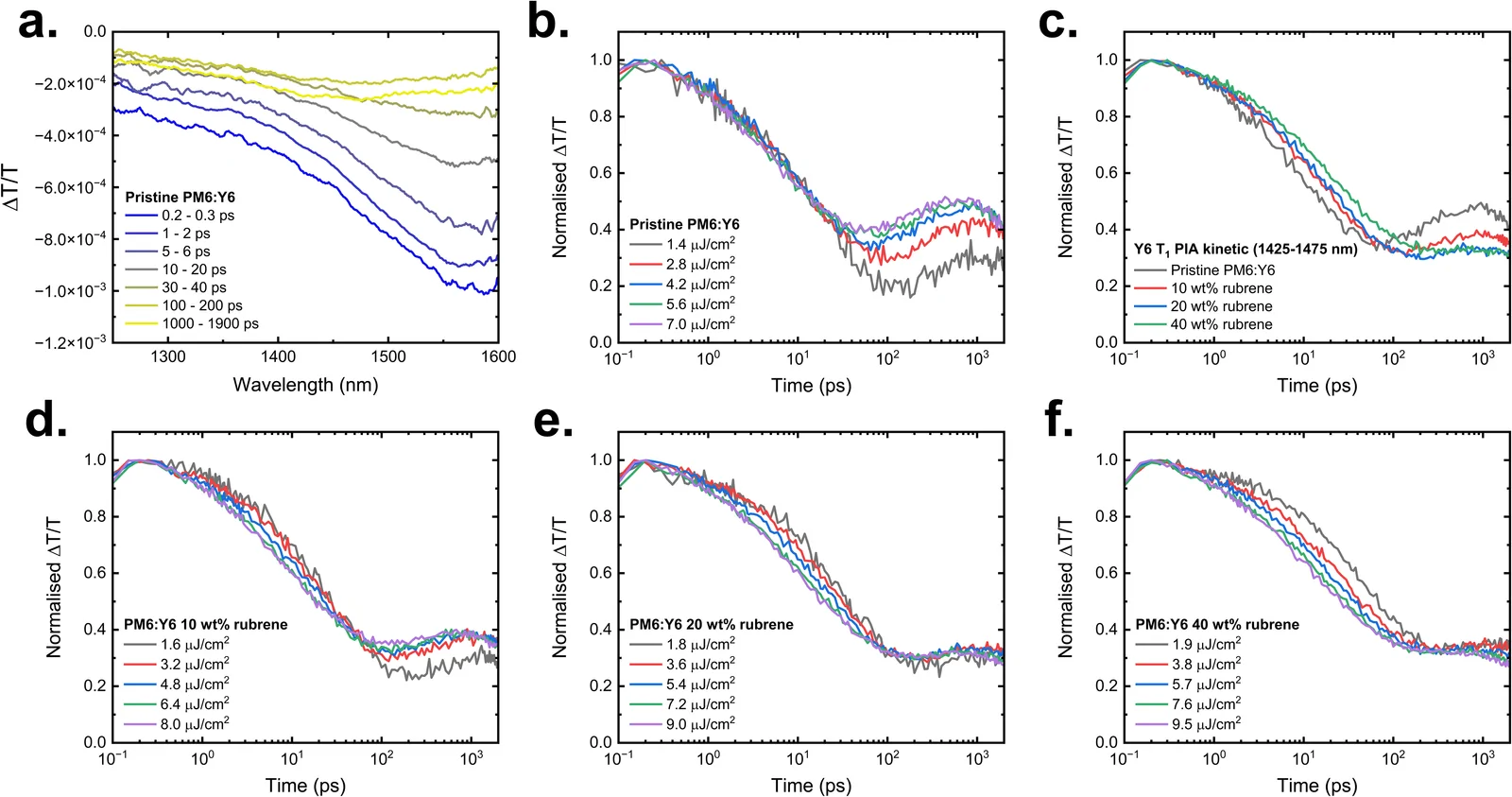
(a) The TA spectra of a pristine PM6:Y6 film measured in the near infrared spectral region. The spectra were acquired with an excitation fluence of 2.8 µJ cm^−2^. (b) The normalised kinetics taken from the Y6 T_1_ PIA region (1425–1475 nm) of a pristine PM6:Y6 film over a fluence series. (c) The normalised kinetics taken from the Y6 T_1_ PIA region (1425–1475 nm) of a pristine PM6:Y6 film and PM6:Y6 films with 10, 20, and 40 wt% rubrene doping. The middle fluence measurement (4.2 to 5.7 µJ cm^−2^) is plotted for each film. (d) The normalised kinetics taken from the Y6 T_1_ PIA region (1425–1475 nm) of a PM6:Y6:rubrene 10 wt% film over a fluence series. (e) The normalised kinetics taken from the Y6 T_1_ PIA region (1425–1475 nm) of a PM6:Y6:rubrene 20 wt% film over a fluence series. (f) The normalised kinetics taken from the Y6 T_1_ PIA region (1425–1475 nm) of a PM6:Y6:rubrene 40 wt% film over a fluence series. All TA measurements were performed with selective excitation of Y6 at 800 nm.

In Fig. 4c, we show a comparison of the normalised kinetics from the Y6 T_1_ PIA region (1425–1475 nm) for the pristine, 10, 20, and 40 wt% films measured with the middle fluence. The normalised TA kinetics for the four other fluences are included in the SI (Fig. S17). To enable a direct comparison between the blends, the fluences have been selected to generate the same initial excitation density on Y6 in all samples (∼2.4 × 10^17^ cm^−3^ in the lowest fluence measurement). This allows us to account for the differences in absorption at 800 nm between the films (Fig. S1) and the increased BHJ thickness with a higher rubrene loading fraction. Curiously, as the rubrene doping fraction is increased, the T_1_ PIA becomes noticeably less intense relative to the point of normalisation (the initial PIA of the photogenerated Y6 hole polarons in the pristine PM6:Y6 film). This indicates a reduction in the population of Y6 T_1_ states in the blend upon the addition of rubrene.

In Fig. 4d–f, we show the TA kinetics from the full fluence series for the Y6 T_1_ PIA (1425–1475 nm) in the 10, 20, and 40 wt% PM6:Y6:rubrene films, respectively. In the 10 wt% rubrene sample, the fluence dependence of the Y6 T_1_ formation kinetic is less prominent compared to pristine PM6:Y6, and this fluence dependence becomes absent in the 20 and 40 wt% rubrene blends. The reduced population of Y6 T_1_ states mentioned above could be explained by either of the two previously described mechanisms (Fig. 3): direct back charge transfer from the PM6:Y6 ^3^CT state to the rubrene T_1_ would avoid the population of the Y6 T_1_, whilst Dexter transfer from the Y6 T_1_ to rubrene would quench the Y6 T_1_ states. However, if the rubrene T_1_ states were only formed *via* direct back charge transfer from the ^3^CT states, we would still expect any Y6 T_1_ states observed in the TA to possess a clear fluence dependence in their formation due to the non-geminate populating mechanism.^11^ Instead, the apparent absence of the expected fluence dependence in the Y6 T_1_ formation for the 20 and 40 wt% rubrene samples implies that the Y6 T_1_ states formed *via* non-geminate recombination are rapidly quenched. Thus, we consider that the apparent fluence-independent Y6 T_1_ formation in the 20 and 40 wt% rubrene blends provides strong evidence that Dexter transfer from the Y6 T_1_ is the dominant pathway for the population of rubrene T_1_ states. However, as it is energetically feasible, we cannot fully rule out a smaller contribution from direct back charge transfer from the ^3^CT state to the rubrene T_1_.

To explore the longer timescale dynamics of the triplet recycling, we have performed nanosecond to microsecond TA studies on the BHJ films. In the IR spectral region (1200–1650 nm), we only observe the Y6 T_1_ PIA on these longer timescales and find that the triplet lifetime decreases as the excitation fluence is increased in a pristine PM6:Y6 film (Fig. S18). This is consistent with the enhanced TCA observed at higher fluences in PM6:Y6.^11^ However, when comparing the pristine PM6:Y6 kinetic to the 10, 20, and 40 wt% rubrene films (Fig. S19), we curiously find an increase in the Y6 T_1_ lifetime, most notable over timescales from tens of nanoseconds to tens of microseconds. The lengthening of the T_1_ lifetime becomes more apparent as the rubrene doping concentration is increased. To investigate this further, we have tracked the decay dynamics of the PM6 hole polaron PIA at 940 nm in the blend (Fig. S20). Here, we also find an increase in the PM6 hole polaron lifetime over similar timescales to the Y6 T_1_ state, and the lifetime again becomes longer with higher rubrene loadings. We note that the enhancement in the triplet and polaron lifetimes in the blend over tens of nanoseconds to tens of microseconds timescales is consistent with the observed timescales of TTA in rubrene in TA measurements performed at comparable excitation fluences.^46^

Based on our observations, we propose that the longer PM6 hole polaron lifetime is linked to the ability of rubrene to exploit TTA to recycle the T_1_ states formed *via* charge recombination back into free charges. As noted previously, the regeneration of charges likely proceeds *via* FRET of the rubrene S_1_ to the PM6 S_1_, followed by electron transfer to Y6. The re-formed PM6 polarons in turn undergo charge recombination again, leading to the generation of more Y6 T_1_ states. As Langevin recombination is already strongly suppressed in PM6:Y6,^47^ this cycle of TTA followed by the re-formation of charges, and recombination to the Y6 T_1_ state, can repeat many times before a terminal recombination event occurs to the ground state.^20^ The consequence of this is a significant increase in the PM6 polaron and Y6 T_1_ lifetimes in the PM6:Y6 blend upon the addition of rubrene, as observed experimentally.

For clarity, we have summarised the proposed mechanism for the TTA recycling in the PM6:Y6:rubrene film in Fig. 3. If the recycling pathway illustrated in Fig. 3 proceeds ideally, TTA can increase the fraction of recombination that ultimately reforms spin-singlet states to 62.5%.^48^ This is based on the spin-statistical argument widely employed in TTA OLEDs, where of the 75% of free electron–hole recombination events initially form spin-triplet states,^12^ of which a maximum of 50% can be converted into singlets *via* TTA. However, in reality, the recombination channels in a real-world OSC device can be much more complicated, which can have a significant impact on the TTA recycling efficiency.^20^

We note that one pathway not yet discussed is the potential for population transfer between the ^1^CT and ^3^CT states. In OSC systems, this coupling is typically provided by the hyperfine interaction (HFI), which drives the periodic oscillation of the CT state population between the ^1^CT and ^3^CT states on timescales of tens to hundreds of nanoseconds.^49^ In general, previous works have considered the HFI too slow to compete with other CT-mediated processes in OSC systems, such as back charge transfer to the molecular triplet, or re-dissociation of the CT state into free charges.^11^ However, if morphological features, such as poorly-connected domains, can support the formation of long-lived and well-separated (electron–hole distance >1 nm) CT states,^17^ then such a pathway is plausible and may impact the triplet recycling processes by modulating the branching ratios between the singlet and triplet recombination channels (Fig. 3).

### 2.4 Morphology characterisation

Having explored the photophysical behaviour of the PM6:Y6:rubrene system, we now turn to the film morphology of the complex ternary blend. To understand how the addition of rubrene impacts the morphology of PM6:Y6, we have characterised the films with grazing incidence wide angle X-ray scattering (GIWAXS), resonant soft X-ray scattering (RSoXS), and atomic force microscopy (AFM). Firstly, to study the molecular packing and orientation of the constituent film components, we have performed GIWAXS measurements on the pristine PM6:Y6 sample and 10, 20, and 40 wt% PM6:Y6:rubrene films. The 2D GIWAXS patterns and 1D azimuthally integrated intensity profiles of pristine PM6, Y6, and rubrene are included in the SI (Fig. S21).

In Fig. 5 we show 2D GIWAXS patterns along with 1D azimuthally integrated intensity profiles of the PM6:Y6:rubrene films. As the film thickness increases with the addition of a higher wt% of rubrene, the 1D profiles have been normalised by the film thickness to enable a more direct comparison between samples. The 2D pattern of pristine PM6:Y6 (Fig. 5a) consists of a scattering feature in the out-of-plane direction (*q*_*z*_ direction in reciprocal space) at *q* = 1.77 Å^−1^ (*d* = 3.56 Å) with a weak shoulder extending to lower *q*. Based on comparison to the pristine films (Fig. S21), we attribute the main peak to a combination of the strong π–π stacking feature of Y6 (*q* = 1.78 Å^−1^; *d* = 3.53 Å) and the weaker π–π stacking signature of PM6 (*q* = 1.70 Å^−1^; *d* = 3.70 Å). The lower-*q* shoulder to the main π–π stacking feature in pristine PM6:Y6 is likely related to the tail of the PM6 π–π stacking feature, which extends into this scattering region. A prominent scattering feature is also visible in the in-plane direction (*q*_*xy*_ direction in reciprocal space) at *q* = 0.305 Å^−1^ (*d* = 20.6 Å), consistent with overlapping scattering from PM6 lamellar stacking and the Y6 (110) reflection. This molecular arrangement is in agreement with GIWAXS measurements of PM6:Y6 films reported elsewhere,^50^ indicating a preferential face-on molecular orientation of both PM6 and Y6.

**Fig. 5:**
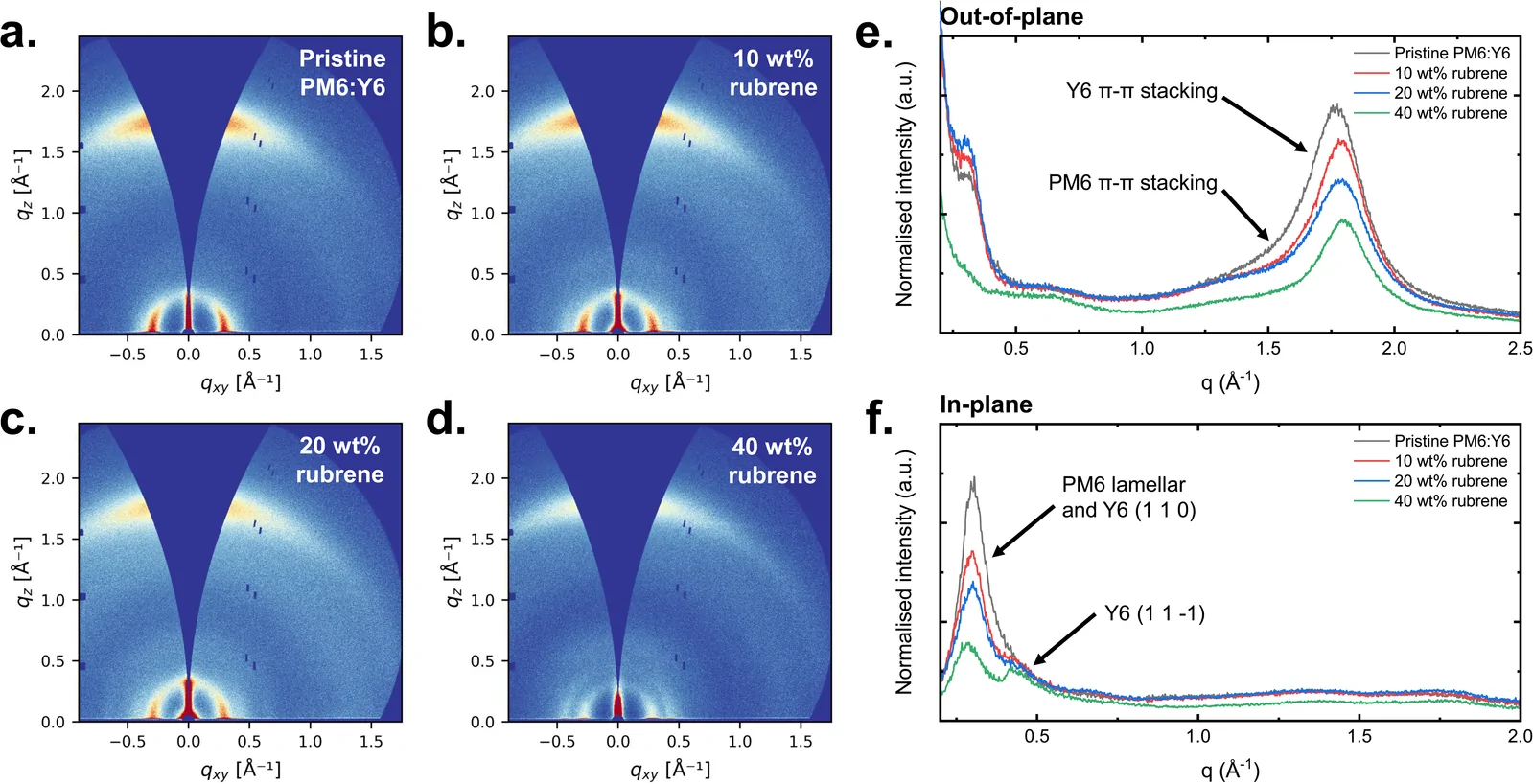
(a) The 2D GIWAXS pattern of a pristine PM6:Y6 film. (b) The 2D GIWAXS pattern of a PM6:Y6 film with 10 wt% rubrene. (c) The 2D GIWAXS pattern of a PM6:Y6 film with 20 wt% rubrene. (d) The 2D GIWAXS pattern of a PM6:Y6 film with 40 wt% rubrene. (e) The 1D azimuthally integrated intensity profile in the out-of-plane direction. (f) The 1D azimuthally integrated intensity profile in the in-plane direction. The 1D azimuthally integrated intensity profiles have been normalised to the film thicknesses of 170, 200, 218, and 233 nm for the pristine PM6:Y6 and 10, 20, and 40 wt% PM6:Y6:rubrene films, respectively.

Upon the addition of rubrene to PM6:Y6 (Fig. 5b–d), the preferential face-on orientation is preserved but the scattering intensity of the out-of-plane Y6 π–π stacking peak (and the in-plane PM6 lamellar/Y6 (110) peak) decreases. This suggests a reduction in the out-of-plane molecular ordering of Y6 (and PM6), consistent with our observation of reduced out-of-plane molecular ordering (*λ*_*Θ*_) in the Y6 T_1_ signal in the PLDMR (Fig. S11 and Table S3). For all rubrene loadings explored here, we do not observe any characteristic scattering features that would be expected for bulk rubrene spin-coated films.^32,51^ This indicates rubrene forms either an amorphous phase or is well intermixed with PM6 and/or Y6. We note that highly crystalline rubrene phases have previously been observed for evaporated rubrene films that have been annealed at higher temperatures (>170 °C) than those used in this work (110 °C).^52^ Interestingly, with the addition of 40 wt% rubrene to the PM6:Y6 film, we find a relative increase in scattering intensity of the previously reported in-plane Y6 (11−1) peak (*q* ∼0.44 Å^−1^) compared to the *q* ∼0.29 Å^−1^ peak, comprising both the PM6 lamellar and Y6 (110) features (Fig. 5d and f).^50^ We note that the two in-plane peaks in the 40 wt% rubrene sample resemble the position and intensities of the (110) and (11−1) features in pristine Y6 (Fig. S21),^53^ potentially indicating a significant loss of PM6 in-plane lamellar ordering. However, due to the similarity in the *q* positions for the PM6 lamellar and Y6 (110) peaks, the overlap between these features prevents a conclusive assignment. Thus, we propose that the addition of large volumes of amorphous rubrene also disrupts both the PM6 and Y6 in-plane molecular ordering, though with a greater impact on PM6.

The atomic force microscopy (AFM) measurement of the pristine PM6:Y6 blend in Fig. 6 shows fibrils associated with Y6,^54^ while no clear PM6 features can be identified. The addition of rubrene does not appear to greatly impact the features observed in the films, with the Y6 fibrils preserved and a similar film roughness found for all samples. The key finding from the AFM analysis is the absence of distinct features corresponding to either rubrene crystallites or aggregates. This strongly suggests that rubrene is present in a mixed PM6:Y6:rubrene phase and supports the GIWAXS observations.

**Fig. 6:**
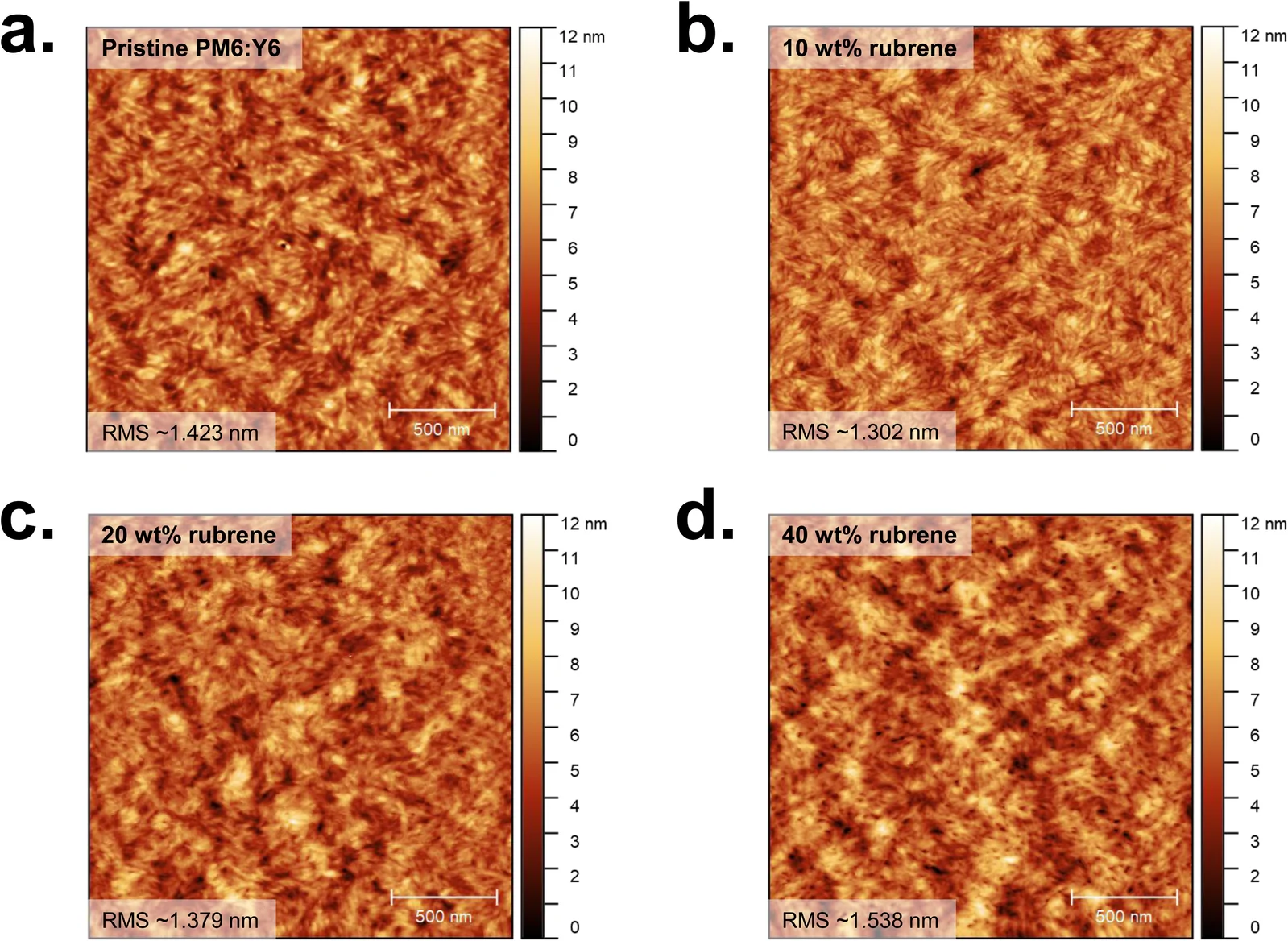
(a) The tapping-mode topography AFM scan of a pristine PM6:Y6 film. (b) The AFM scan of a PM6:Y6 film with 10 wt% rubrene. (c) The AFM scan of a PM6:Y6 film with 20 wt% rubrene. (d) The AFM scan of a PM6:Y6 film with 40 wt% rubrene.

To understand how the addition of rubrene impacts the phase separation of the blend films, RSoXS measurements were performed. In RSoXS, domains of different material composition and scattering contrasts give rise to features in the scattering intensity corresponding to characteristic length scales associated with phase-separation. In Fig. S22, the energy-dependent integrated scattering intensity for the PM6:Y6:rubrene compositions studied in this work shows that scattering contrast is indeed dominated by differences in domain composition. The RSoXS measurement of the pristine PM6:Y6 film in Fig. 7 is featureless and reveals that the films are not strongly phase separated, indicating mostly intermixed PM6 and Y6, as reported previously.^55^ Due to the chemical similarity between PM6 and Y6, the material contrast between the pure, ordered Y6 and PM6 domains observed with GIWAXS and AFM, and the intermixed PM6:Y6 domains, is too weak to yield a distinct scattering feature in RSoXS.

**Fig. 7:**
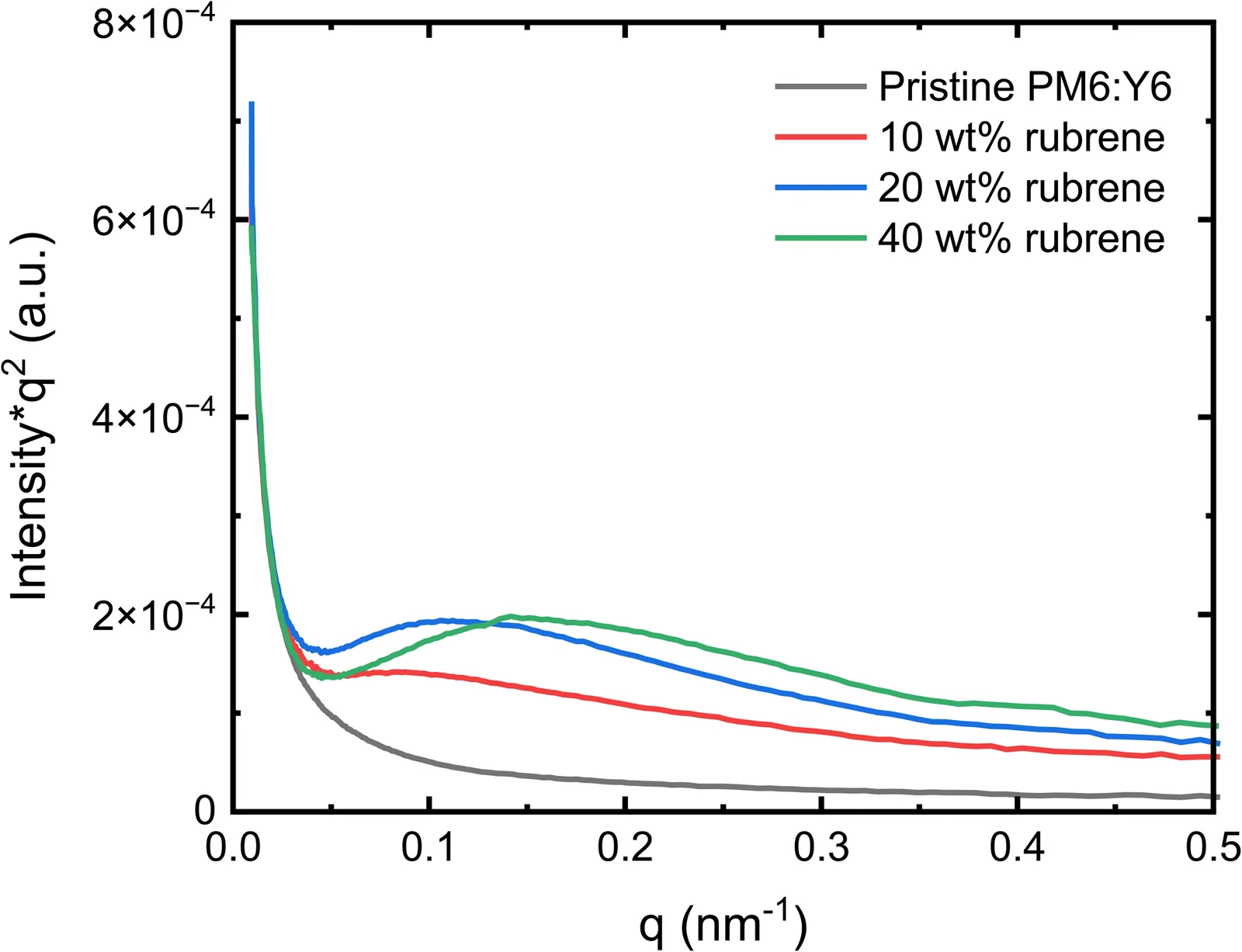
The mass-roughness normalised RSoXS profiles of a pristine PM6:Y6 film, and PM6:Y6 films with 10, 20, and 40 wt% rubrene. The RSoXS profiles are taken at resonance (284 eV).

Upon addition of 10 wt% rubrene to PM6:Y6, a broad feature appears at *q* = 0.08 nm^−1^ (*d* = 79 nm), indicative of phase separation. With increasing rubrene content, this feature grows in intensity and shifts to larger *q*-values, *e.g.*, for 40 wt% rubrene *q* = 0.15 nm^−1^ (*d* = 42 nm). Attributing RSoXS scattering features to specific materials in ternary blends is challenging and prevents a more quantitative analysis. However, since GIWAXS and AFM data show pure Y6 and PM6 phases, while also suggesting an intermixed PM6:Y6:rubrene phase, we propose that the scattering arises from the material contrast between the pure PM6 and Y6, and the (ternary) intermixed domains. The chemical differences of rubrene compared to PM6 and Y6 cause a stronger material contrast and hence more pronounced scattering features than the pristine PM6:Y6 blend. The increased rubrene content then naturally enhances the scattering intensity, with the peak shift indicating reduced spacing between domains. This is consistent with the decrease in ordered Y6 and PM6 domains observed in GIWAXS.

In summary, the GIWAXS, AFM, and RSoXS measurements, supported by observations in the PLDMR, form a coherent picture. The pristine PM6:Y6 blend mainly consists of ordered domains of Y6, evidenced by the corresponding peaks in the GIWAXS and the characteristic Y6 fibril structure observed in the AFM, as well as amorphous intermixed domains of PM6:Y6, evidenced by the absence of scattering features in the RSoXS. Upon the addition of rubrene to the PM6:Y6 blend, GIWAXS shows no qualitative change in preferential orientation, but a successive reduction of ordered Y6 and PM6 domains. The RSoXS data confirms such a multiphase system with scattering likely arising due to the contrast between pure PM6 and Y6, and the intermixed PM6:Y6:rubrene domains. Altogether, the microstructure data suggests that rubrene does not phase separate to form pure rubrene domains but is instead located within mixed PM6:Y6:rubrene domains. While it is less clear how much rubrene–rubrene contact is present in these mixed domains, our photophysical studies suggest that it is sufficient to enable TTA. We believe that the close contact between rubrene and Y6 in the mixed domains, as well as the proximity of rubrene to the interface between PM6 and Y6 where the formation of Y6 T_1_ states *via* non-geminate recombination takes place, is essential for enabling the triplet recycling we observe in the PM6:Y6:rubrene system.

### 2.5 Organic solar cell device studies

To complete our study, we have fabricated PM6:Y6:rubrene OSC devices to understand the impact of adding rubrene on the device function. An inverted device architecture was used, consisting of the following device stack: ITO/ZnO/BHJ/MoO_*x*_/Ag. The current density–voltage (*J*–*V*) curves for the PM6:Y6:rubrene devices are shown in Fig. 8, with a summary of the device performance parameters presented in Table 1. The reference pristine PM6:Y6 device shows reasonable performance, with a PCE of 13.9% obtained for the champion device. The PCE of PM6:Y6 we obtain here may be lower than some other reports as it is known that the PCE depends on the molecular weight of PM6-derivatives.^56,57^ Nonetheless, upon the addition of 10 wt% rubrene, we observe an increase in the PCE to 14.1%, mainly through an improvement in the short circuit current density (*J*_SC_) of the device from 23.05 to 24.21 mA cm^−2^. The fill factor (FF) of the 10 wt% device decreases slightly from 74.0% to 72.6%, which we attribute to the increased thickness of the BHJ layer of the PM6:Y6:rubrene devices, whilst the *V*_OC_ remains nearly unchanged. Upon the addition of 20 and 40 wt% rubrene, the PCE drops slightly to 13.52% and 12.03%, respectively. This can be primarily ascribed to a further reduction in the FF, again likely related to the increasing BHJ thickness. The *J*_SC_ is also reduced to 21.79 mA cm^−2^ in the device with 40 wt% rubrene, which is potentially related to the slower hole transfer process from Y6 to PM6 in the rubrene-containing blends (Fig. S23) that is known to decrease the charge generation efficiency.^57,58^ Thus, it appears that the accumulation of large amounts of rubrene at the interface between PM6 and Y6 in the mixed phase may negatively affect the hole transfer process. However, it is noteworthy that a device with such a high rubrene loading wt% can still operate so efficiently. This points to the relatively good tolerance of the PM6:Y6 BHJ to the addition of even very large quantities of rubrene.

**Fig. 8:**
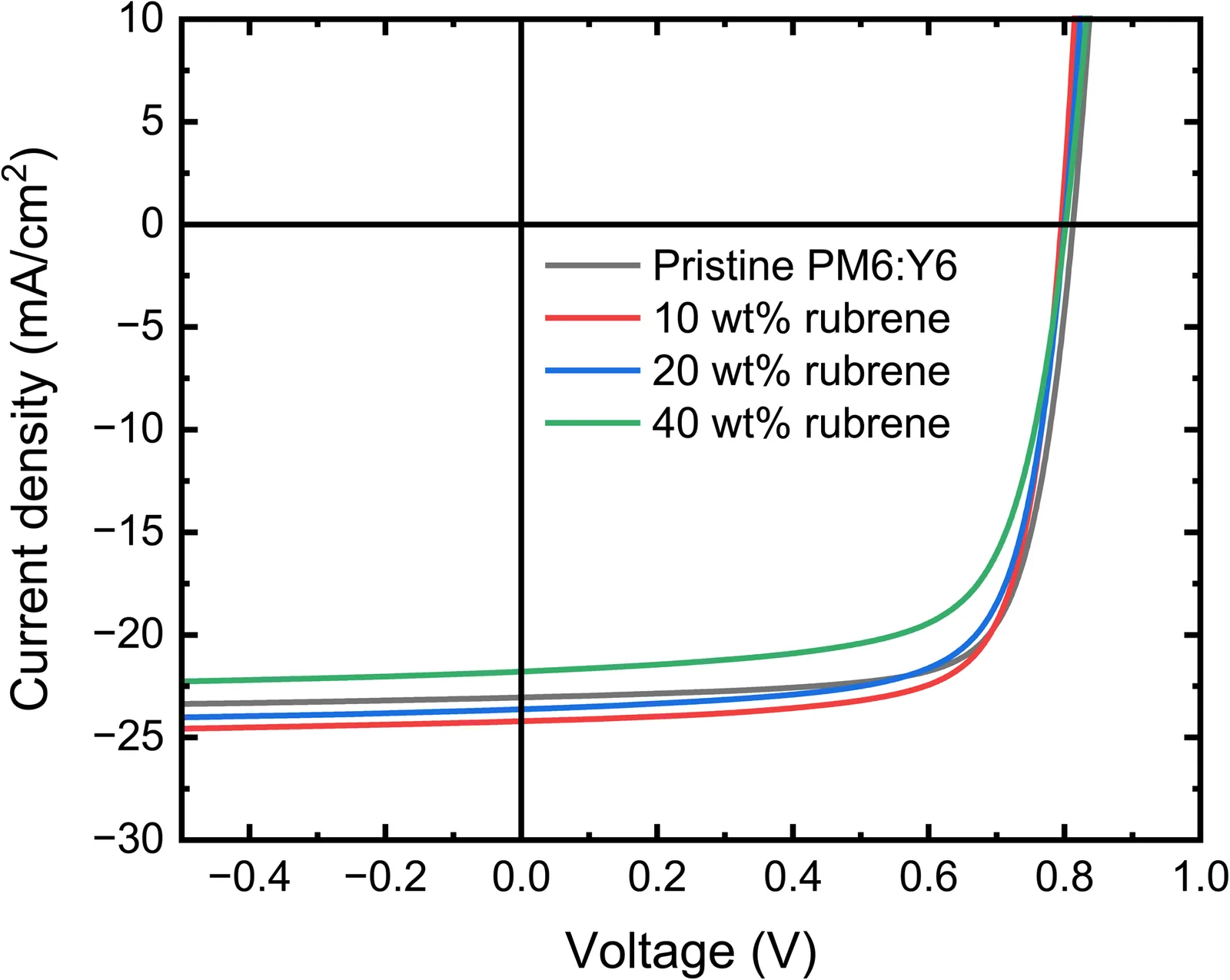
The current density–voltage curves of the champion pristine PM6:Y6 OSC device, and the champion PM6:Y6 devices with 10, 20, and 40 wt% rubrene.

**Table 1:** The OSC device performance parameters of the pristine PM6:Y6 and PM6:Y6 blends with 10, 20, and 40 wt%. The value for the champion cell is given, with the average and standard deviation of four individual devices provided in brackets

Device	*V*_OC_ (V)	*J*_SC_ (mA cm^−2^)	FF (%)	PCE (%)
Pristine PM6:Y6	0.81	23.05 (22.74 ± 0.25)	74.0 (74.2 ± 0.3)	13.90 (13.72 ± 0.13)
PM6:Y6 with 10 wt% rubrene	0.80	24.21 (24.39 ± 0.18)	72.6 (72.0 ± 0.5)	14.10 (14.01 ± 0.07)
PM6:Y6 with 20 wt% rubrene	0.80	23.63 (23.56 ± 0.06)	71.1 (70.8 ± 0.3)	13.52 (13.39 ± 0.11)
PM6:Y6 with 40 wt% rubrene	0.80	21.79 (21.59 ± 0.16)	68.5 (67.9 ± 0.5)	12.03 (11.77 ± 0.20)

To understand the factors behind the increase in *J*_SC_ in the PM6:Y6 device upon the addition of moderate amounts of rubrene, we examine the photovoltaic external quantum efficiency (EQE) curves of the OSCs (Fig. S24). To enable the comparison more clearly, we have plotted the change in EQE as a function of wavelength between the PM6:Y6:rubrene and the reference pristine PM6:Y6 devices (Fig. S25). For the 10 wt% rubrene devices, the EQE generally increases over the 470–950 nm region that covers the main absorption bands of PM6 and Y6. However, the trends are less clear below 470 nm: between 400–470 nm, there is a sharp decrease in the relative EQE, whilst there is a large increase between 315–400 nm. As there is little difference between the absolute absorption of the active layers in the 315–470 nm spectral region (Fig. S1), it is instead likely that the change in EQE here is related to differences in optical interference within the device caused by changes in the active layer thickness (170 *vs.* 200 nm for pristine PM6:Y6, and 10 wt% rubrene devices, respectively).^59^

Indeed, this raises the question of whether the increase in EQE seen between 470–950 nm is a direct result of triplet recycling by rubrene or could also be attributed to differences in optical interference within the device. Though the impact of non-geminate recombination at short circuit is likely to be small for a good OSC device,^60^ previous reports have suggested that significant recombination to the T_1_ state in an OSC device can negatively impact the *J*_SC_ as T_1_ recombination can outcompete charge extraction.^15,61,62^ Thus, triplet recycling by rubrene, which has the effect of increasing the charge carrier lifetime in the blend (Fig. S20b), may provide an opportunity for photocurrent to be extracted from excitations that would otherwise be lost *via* terminal recombination to the T_1_ state on Y6. This could manifest as a slightly improved photocurrent generation in the PM6:Y6 device with 10 wt% rubrene. However, due to the uncertainty related to the likely differences in optical interference between the pristine PM6:Y6 and 10 wt% rubrene devices, it is difficult to quantify how much, if any, of the increase in *J*_SC_ is due to triplet recycling in this case.

One of the key benefits of triplet recycling *via* TTA is the potential to obtain light emission from the otherwise dark T_1_ states generated following non-geminate charge recombination. This, in theory, should result in an improvement in the *V*_OC_ by enhancing the radiative recombination in an OSC device.^20^ However, in our PM6:Y6:rubrene devices, there is no improvement in *V*_OC_ compared to the reference pristine PM6:Y6 device. This has led us to further consider the limitations of engineering TTA into an OSC device. As outlined in our previous work, the potential benefit to the *V*_OC_ from TTA depends on what fraction of the re-formed S_1_ states subsequently generate charges *versus* directly undergoing radiative decay.^20^ We note that this leads to an important trade-off between *V*_OC_ and *J*_SC_: the larger the fraction of regenerated S_1_ states that go on to re-form charges, the greater the potential improvement in *J*_SC_. However, efficient charge generation from the re-formed S_1_ states reduces the fraction of these S_1_ states that can undergo radiative recombination. This minimises the potential gain in *V*_OC_ as each pass through the cycle shown in Fig. 3 halves the number of S_1_ states formed following TTA as two rubrene T_1_ states can, at most, form one rubrene S_1_ state. In the case of the PM6:Y6:rubrene devices reported here, the presence of efficient FRET from rubrene to PM6, followed by charge transfer, represents a dominant channel for the eventual re-formation of free charges from the regenerated rubrene S_1_ states (Fig. 3). Thus, we consider that the PM6:Y6:rubrene system represents the extreme where the regeneration of charges is maximised at the expense of radiative recombination. Therefore, the PM6:Y6:rubrene OSC devices show an enhanced *J*_SC_ compared to the reference pristine PM6:Y6 device, though we stress it is not possible to disentangle the contributions to *J*_SC_ from triplet recycling and changes in the optical interference in the device, but no improvement in *V*_OC_.

## 3. Conclusion

In our work, we have demonstrated a new tactic to mitigate losses *via* the T_1_ state in an OSC device. This has been achieved by introducing an extrinsic TTA material into the BHJ, which is able to accept the T_1_ states formed *via* non-geminate recombination and ultimately recycle them back into free charges. Working with the archetypal TTA material rubrene and the model OSC system PM6:Y6, we have used PLDMR measurements to show that the rubrene T_1_ state directly modulates the NIR luminescence in the PM6:Y6:rubrene ternary system. Using TA spectroscopy, we have further revealed that the primary T_1_ recycling mechanism involves the transfer of T_1_ states from Y6 to rubrene. The T_1_ states on rubrene then undergo TTA to generate rubrene S_1_ states, which likely transfer to PM6 *via* FRET, followed by charge generation with Y6. Our detailed morphological characterisation of the PM6:Y6:rubrene system uncovers a complex evolution of the blend morphology upon the addition of rubrene. This primarily involves the formation of mixed domains containing PM6, Y6, and rubrene. We believe that the presence of mixed domains containing all three components enables the transfer of T_1_ states formed on Y6 *via* non-geminate charge recombination to rubrene and thus underpins the triplet recycling process.

We believe that our study provides a useful proof of concept for the capability of an extrinsic TTA dopant to mitigate T_1_-induced losses in OSCs. However, our work raises several key areas that require attention in future studies. For example, the evolution of the blend morphology upon the addition of the extrinsic TTA dopant plays a key role in controlling the triplet recycling process. We believe it is essential that the TTA dopant shows good miscibility with both the donor and acceptor materials so that it can localise near the donor:acceptor interface to accept the T_1_ states formed *via* non-geminate charge recombination. However, care must be taken to avoid the accumulation of too much rubrene at the interface as this can negatively impact the charge transfer process and reduce the *J*_SC_. Furthermore, more work is required to understand the factors controlling the trade-off between improving the *V*_OC_ and *J*_SC_, with the former benefitting from the direct radiative recombination of S_1_ states generated *via* TTA,^20^ whilst the latter is improved when the S_1_ states proceed to re-form free charges. One potential tactic could involve modulating the overlap of the TTA dopant PL with the absorption spectra of the electron donor (and electron acceptor) in the blend. An enhanced spectral overlap will result in efficient FRET to the donor material, followed by charge generation, as observed in our work. By contrast, a reduced spectral overlap can result in the generation of efficient luminescence directly from the TTA dopant itself.^24^ To this end, we propose that moving beyond rubrene to explore the wide variety of TTA dopants reported in the literature may prove to be a fruitful endeavour in the quest to optimise both the morphology and the balance between improving the *V*_OC_ and *J*_SC_. Finally, the deeper mechanistic and morphological understanding provided by our work will also benefit the development of more efficient solid-state TTA upconversion systems, which face many of the same challenges as triplet recycling in OSCs,^63^ and can also employ OSC-based donor:acceptor blends as triplet sensitisers.^24,25^

## 4 Methods

### 4.1. Film and organic solar cell device fabrication

PM6 and Y6 were purchased from Solarmer, and rubrene was purchased from Sigma-Aldrich. All materials were used as received. OSC devices were fabricated with an inverted structure: ITO/ZnO/BHJ/MoO_*x*_/Ag. A 40 nm ZnO nanoparticle film was spin coated onto the clean ITO coated substrate and then annealed at 120 °C for 30 minutes. For the pristine PM6:Y6 BHJ layer, a total solid concentration of 15.4 mg mL^−1^ in chloroform with 0.5 vol% of 1-chloronaphthalene (1-CN) was used. The donor : acceptor ratio was 1 : 1.2. For the PM6:Y6:rubrene BHJ layers, rubrene was added to the PM6:Y6 solution so that the total concentration increased above 15.4 mg mL^−1^. Rubrene loadings are expressed as a percentage of the combined mass of PM6 and Y6, excluding rubrene. For example, for PM6:Y6 with 20 wt% rubrene, 3.08 mg mL^−1^ of rubrene was added to the 15.4 mg per mL PM6:Y6 solution, giving a total solution concentration of 18.48 mg mL^−1^. The active layer was spin coated at 3000 rpm followed by the thermal annealing at 110 °C for 10 minutes. Subsequently, the films were transferred to an evaporator chamber which maintained a vacuum of 2 × 10^−6^ mbar. A 15 nm layer of MoO_3_ followed by 100 nm of Ag were evaporated on top of the active layer, giving an active area of 3.43 mm^2^ for each sub cell on the device.

Thin film samples for optical measurements were prepared with the same solutions and treatments for device fabrication on quartz substrates.

All the OSC devices and films were fabricated in a nitrogen-filled glove box.

### 4.2 Organic solar cell device characterisation

*J*–*V* characteristics were measured using an Oriel LSH-7320 ABA LED solar simulator with an intensity of 100 mW cm^−2^, calibrated by a standard silicon cell. *J*–*V* curves were recorded with a Keithley 2400 digital source meter using a custom LabVIEW code.

The EQE measurements were performed using the Spectral Response Measurement System QE-R3011 (EnliTechnology Co., Ltd). A calibrated silicon detector provided by the Enli Technology was used to determine the absolute photosensitivity at different wavelengths.

### 4.3 Photoluminescence detected magnetic resonance

PLDMR experiments were carried out with a modified X-band spectrometer (Bruker E300) equipped with a continuous-flow helium cryostat (Oxford ESR 900) and a microwave cavity (Bruker ER4104OR, ∼9.43 GHz) with optical access. Optical irradiation was provided by either a 532 nm (Cobolt Samba CW 532 nm DPSSL) or a 785 nm (Coherent OBIS LX 785 nm) continuous wave laser using a side opening of the cavity. The PL was detected with a silicon photodiode (Hamamatsu S2281) on the opposite opening, using either a 561 or 830 nm long-pass filter to reject the excitation light. The PL signal was amplified by a current/voltage amplifier (Femto DHPCA-100). The PL was recorded by a lock-in detector (Ametek SR 7230), referenced by on-off modulating the microwaves with a modulation frequency of 547 Hz. The microwaves were generated with a microwave signal generator (Anritsu MG3694C), amplified to 3 W (microsemi) and guided into the cavity.

### 4.4 Transient absorption spectroscopy

TA measurements were performed on a setup powered using a commercially available Ti:sapphire amplifier (Spectra Physics Solstice Ace). The amplifier operates at 1 kHz and generates 100 fs pulses centred at 800 nm with an output of 7 W. For the picosecond TA measurements, a portion of the laser fundamental was used for sample excitation at 800 nm. The probe pulse was generated using home-built non-colinear optical parametric amplifiers (NOPAs) in the visible (475–530 nm) and IR (1250–1600 nm) spectral regions. For nanosecond TA measurements in the NIR (850–1000 nm), the probe pulse was generated using a home-built NOPA, whilst the pump at 532 nm was provided by the second harmonic of an electronically-gated nanosecond pulsed Nd:YVO_4_ laser (Innolas Picolo MOPA 25). For the nanosecond TA measurements in the IR (1200–1650 nm), a portion of the laser fundamental was used for sample excitation at 800 nm, whilst the probe pulse was provided by a low timing jitter nanosecond pulsed supercontinuum laser (LEUKOS DISCO STM-1-UV). For the nanosecond TA measurements, an electronic delay generator (Stanford Research Systems DG645) was used to control the time delay between the pump and probe.

For TA measurements <1000 nm, the probe pulses are collected with a Si dual-line array detector (Hamamatsu S8381-1024Q). For TA measurements >1000 nm, the probe pulses are collected with an InGaAs dual-line array detector (Hamamatsu G11608-512DA). Both detectors are driven and read out by custom-built boards from Stresing Entwicklungsbüro. The probe light was split into two identical beams by a 50/50 beamsplitter. This allowed for the use of a second reference beam which also passes through the sample but does not interact with the pump. The role of the reference was to correct for any shot-to-shot fluctuations in the probe that would otherwise greatly increase the structured noise in our experiments.

### 4.5 GIWAXS

GIWAXS measurements were performed using a Xeuss 2.0 laboratory beamline (Xenocs) equipped with a liquid gallium MetalJet source (Excillum), producing X-rays with an energy of 9.243 keV (*λ* = 1.34 Å). A collimated X-ray beam was directed at sample surfaces inclined at a grazing angle of 0.15° and scattered X-rays were detected by a Pilatus3R 1M 2D X-ray detector (Dectris) positioned ∼300 mm from the sample centre. The sample to detector distance was calibrated using a silver behenate standard in transmission geometry. During measurement, the entire flight path including collimation tubes and sample chamber was held under vacuum to minimise background air scatter. GIWAXS data were corrected, reshaped and reduced using code based on pyFAI and pygix Python libraries.^64^ 1D intensity profiles were generated by azimuthally integrating the 2D patterns as a function of *q* = 4π sin *θ*/*λ* where 2*θ* is the angle between the incident and scattered X-ray of wavelength *λ*. Integrations were performed across the full *q* range through various azimuthal (*χ*) angles normal to the incident beam on the detector; out-of-plane (−20° < *χ* < 20°) and in-plane (20° < *χ* < 90°). The 1D intensity profiles were normalised to the film thickness of each sample to offer a better comparison between scattering intensities.

### 4.6 AFM

All AFM height images were obtained using a Bruker Multimode 8 atomic force microscope in tapping mode under nitrogen atmosphere. AFM tips with a force constant of 40 N m^−1^ and resonance frequency of *ca.* 300 kHz were used in the AFM measurements. First-order image flattening was performed on the morphology images and the measured morphology heights were offset to set the scale start point to 0 nm.

### 4.7 RSoXS

RSoXS measurements were conducted at beamline 11.0.1.2 of the Advanced Light Source (ALS) at Lawrence Berkeley National Laboratory. A photon energy of 284 eV was chosen to enhance material contrast while minimising sample damage. The blend films were coated on PSS-coated Si substrates and then transferred by floating on water onto a 2 mm × 2 mm, 100 nm thick Si_3_N_4_ membrane, supported by a 5 mm × 5 mm, 200 µm thick Si frame (Norcada Inc.). Data collection was performed in a transmission geometry using linearly polarised photons under high vacuum (1 × 10^−7^ torr). A Peltier-cooled CCD detector (Princeton PI-MTE) at −45 °C was used to capture the 2D scattering patterns. The sample-detector distance was calibrated using a PS300 standard (Sigma-Aldrich).

## Author contributions

A. J. G. conceived the work. D. M. L. U. and A. J. G. performed the TA measurements. D. M. L. U. measured the steady state absorption and photoluminescence. R. C. K. and D. T. W. T. carried out the GIWAXS and analysed the data. J. G. performed the PLDMR measurements. P. K. and S. M. ran the RSoXS measurements and analysed the data. S. Y. conducted the AFM measurements. N. J. fabricated and tested the OSC devices. H. Y. fabricated the Y6:rubrene bilayer samples. J. M. F., H. A., T.-Q. N., A. S., V. D., N. C. G., and A. J. G. supervised their group members involved in the project. A. J. G. wrote the manuscript with input from all authors. All authors read and provided feedback on the manuscript prior to submission.

## Conflicts of interest

The authors declare no competing financial interest.

## Supplementary Material

EL-OLF-D6EL00075D-s001

## Data Availability

The data that supports the plots within this paper are available at the Zenodo repository. DOI: 10.5281/zenodo.23189384. Supplementary information (SI) is available. See DOI: https://doi.org/10.1039/d6el00075d.
