# Supplementary material for: Triplet exciton recycling in organic solar cells enabled by an extrinsic triplet–triplet annihilation dopant
Source: EES Solar. 2026 Oct 12. Online ahead of print. doi: 10.1039/d6el00075d (PMC13656303; doi:10.1039/d6el00075d)
Supplement: Supplementary file 1 [file EL-OLF-D6EL00075D-s001.pdf]

Supplementary Information (SI) for EES Solar.
This journal is © The Royal Society of Chemistry 2026

Supplementary Information for

Triplet exciton recycling in organic solar cells enabled by an extrinsic triplet-triplet annihilation dopant

Darcy M. L. Unson[1#], Rachel C. Kilbride[2,3,4#], Jeannine Grüne[1,5], Daniel T. W. Toolan[6], Pascal Kaienburg[7], Subhrangsu Mukherjee[8], Sangcheol Yoon[9], Nakul Jain[10], Hanbo Yang[11], Jarvist M. Frost[11], Harald Ade[8], Thuc-Quyen Nguyen[9], Andreas Sperlich[5], Vladimir Dyakonov[5], Neil C. Greenham[1], and Alexander J. Gillett[1,10]*.

[1]Cavendish Laboratory, University of Cambridge, J J Thomson Avenue, Cambridge, CB3 0HE, United Kingdom
[2]School of Mathematics and Physical Sciences, University of Sheffield, Brook Hill, Sheffield, S3 7HF, United Kingdom
[3]Department of Physics, The University of Warwick, Coventry, CV4 7AL, United Kingdom
[4]XMaS, The UK X-ray Materials Science Facility, European Synchrotron Radiation Facility, F-38043 Grenoble, France
[5]Experimental Physics 6, Julius Maximilian University of Würzburg, Am Hubland, Würzburg 97074, Würzburg, Germany
[6]Department of Materials, University of Manchester, Booth St East, Manchester, M13 9SS, United Kingdom
[7]Clarendon Laboratory, Department of Physics, University of Oxford, Parks Road, OX1 3PU Oxford, United Kingdom
[8]Department of Physics and Organic and Carbon Electronics Laboratories (ORaCEL), North Carolina State University, Raleigh, NC 27695, USA
[9]Centre for Polymers and Organic Solids, Department of Chemistry and Biochemistry, University of California at Santa Barbara, Santa Barbara, CA 93106, USA
[10]Department of Physics, Chemistry and Biology (IFM), Linköping University, 581 83 Linköping, Sweden
[11]Department of Physics, Imperial College London, London, SW7 2BW, United Kingdom

[#]Equal contribution

*Corresponding author. Email: alexander.gillett@liu.se

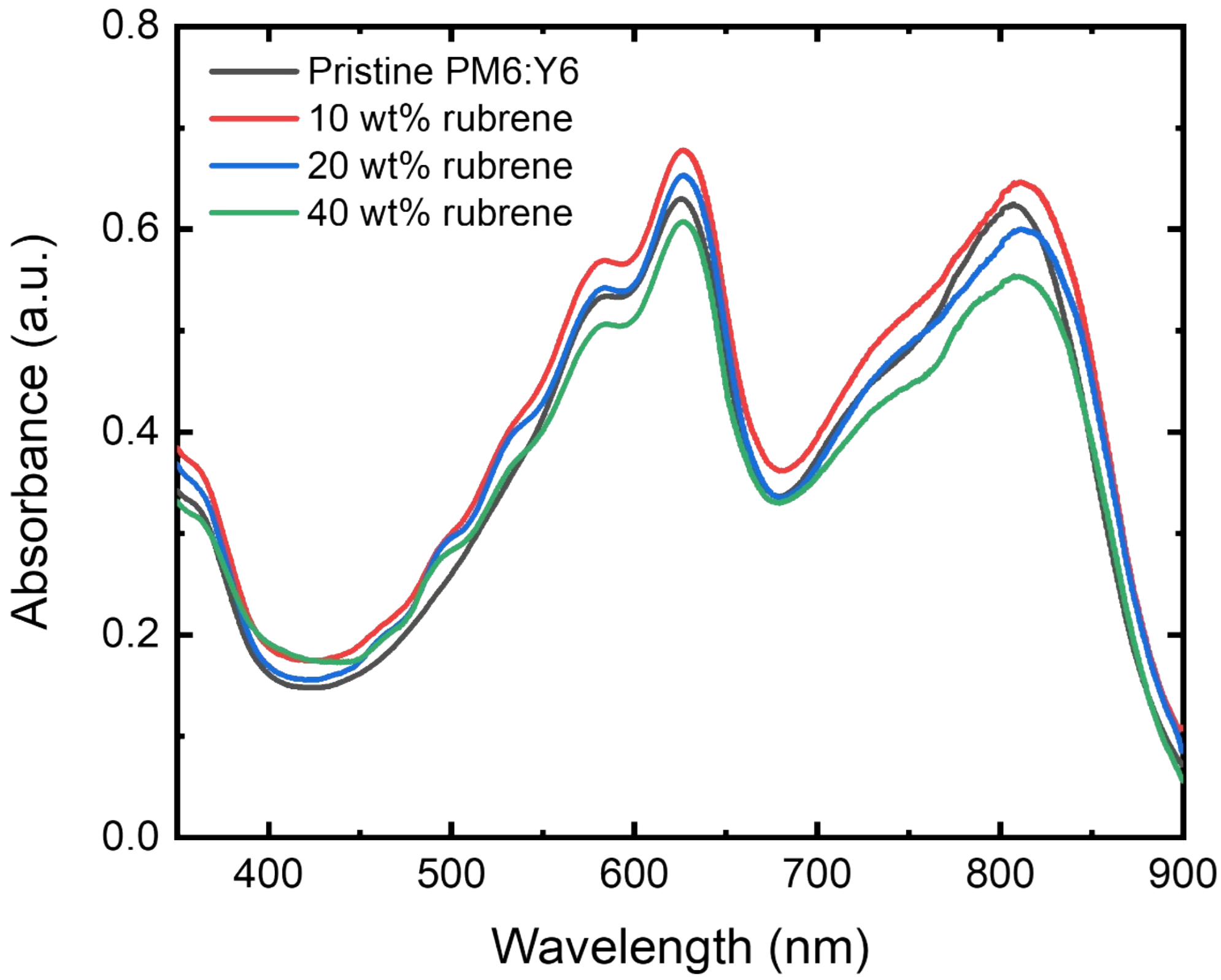

Figure S1. The unnormalised steady-state absorption spectrum of the pristine PM6:Y6 film, as well as PM6:Y6 films doped with 10, 20, and 40 wt% rubrene.

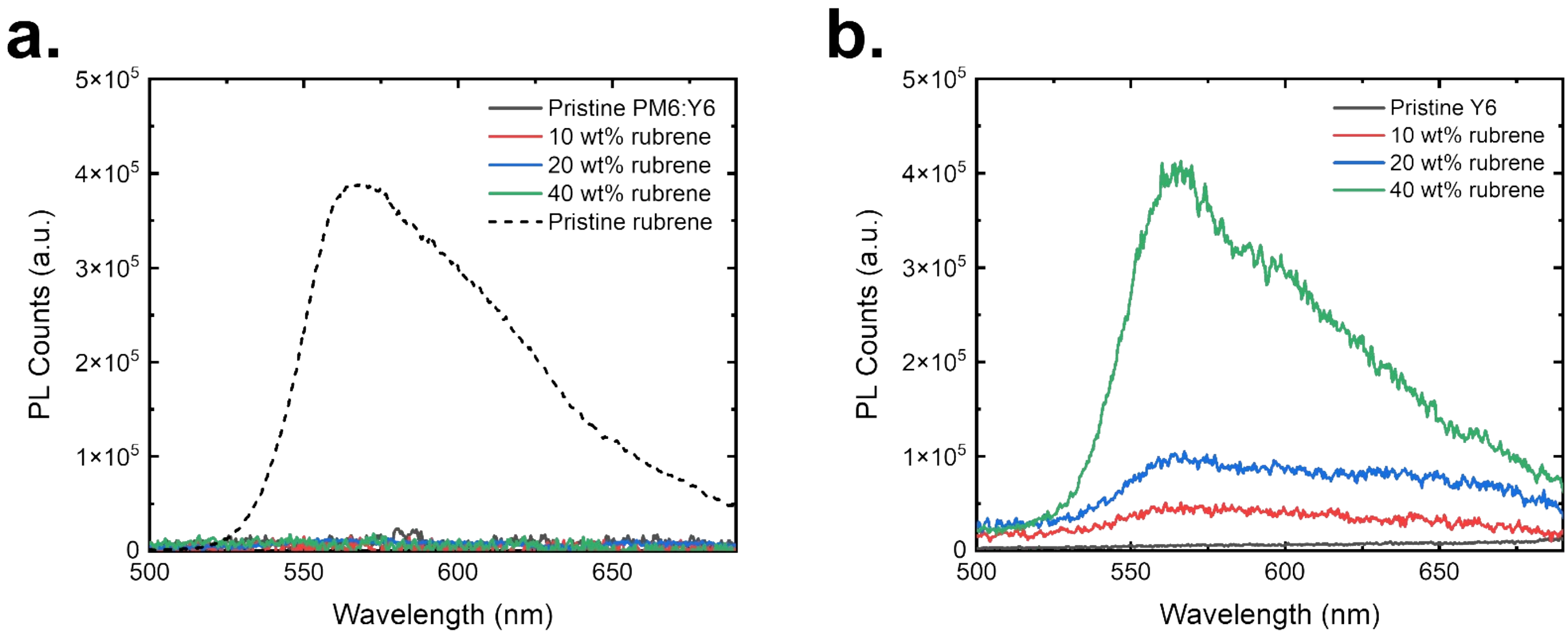

Figure S2. a. The steady-state photoluminescence spectra of a pristine PM6:Y6 film, as well as PM6:Y6 films doped with 10, 20, and 40 wt% rubrene. The steady-state photoluminescence spectrum of a pristine rubrene film has also been included for reference. **b.** The steady-state photoluminescence spectra of a pristine Y6 film, as well as Y6 films doped with 10, 20, and 40 wt% rubrene. The excitation wavelength for the pristine PM6:Y6, Y6, PM6:Y6:rubrene, and Y6:rubrene blend samples was 800 nm to avoid any direct excitation of rubrene. The pristine rubrene film was excited at 532 nm.

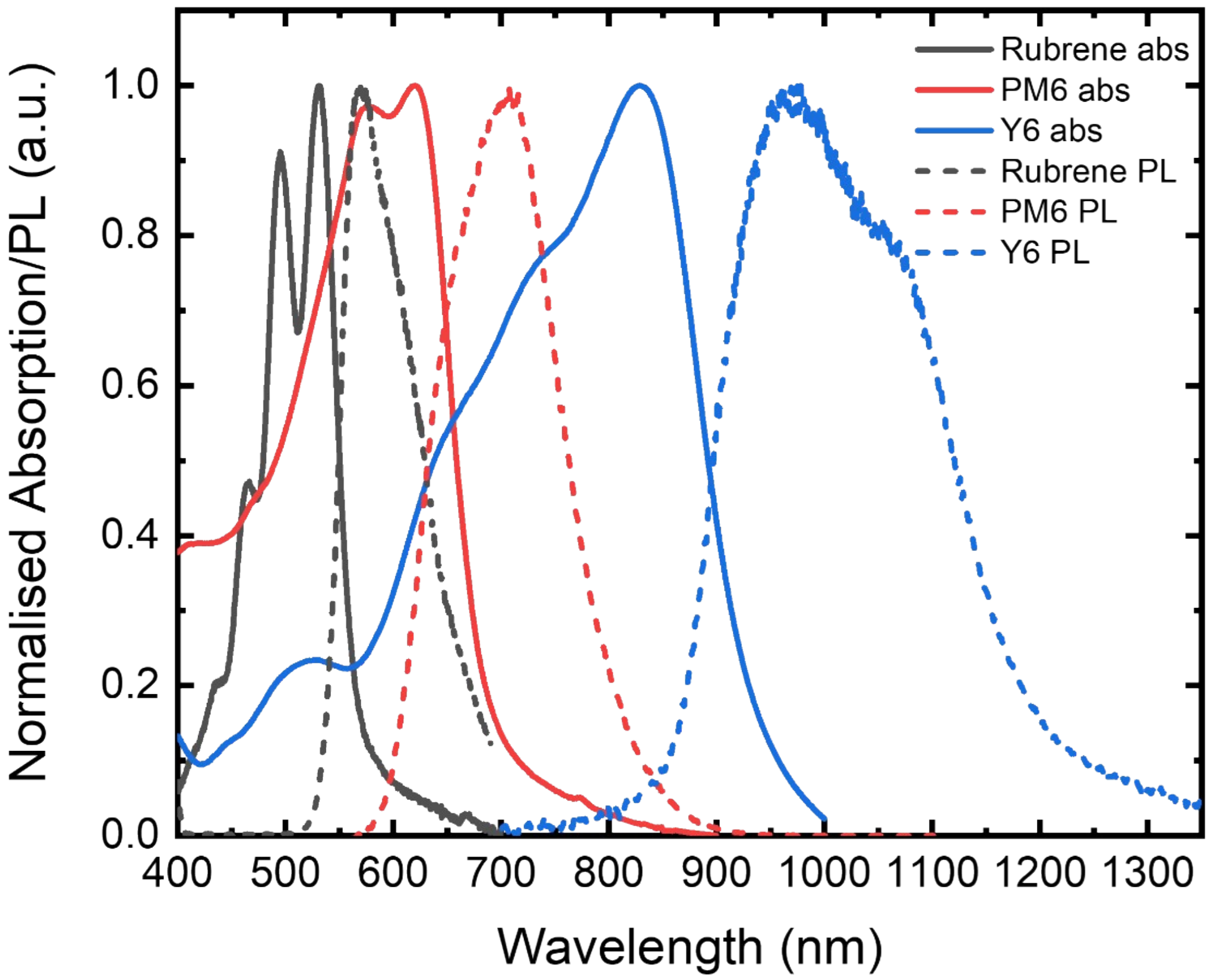

Figure S3. The steady-state absorbance spectra of pristine rubrene, PM6, and Y6 films, with the steady-state photoluminescence spectra of the same samples overlaid. The rubrene film was excited at 405 nm, the PM6 film at 532 nm, and the Y6 film at 658 nm.

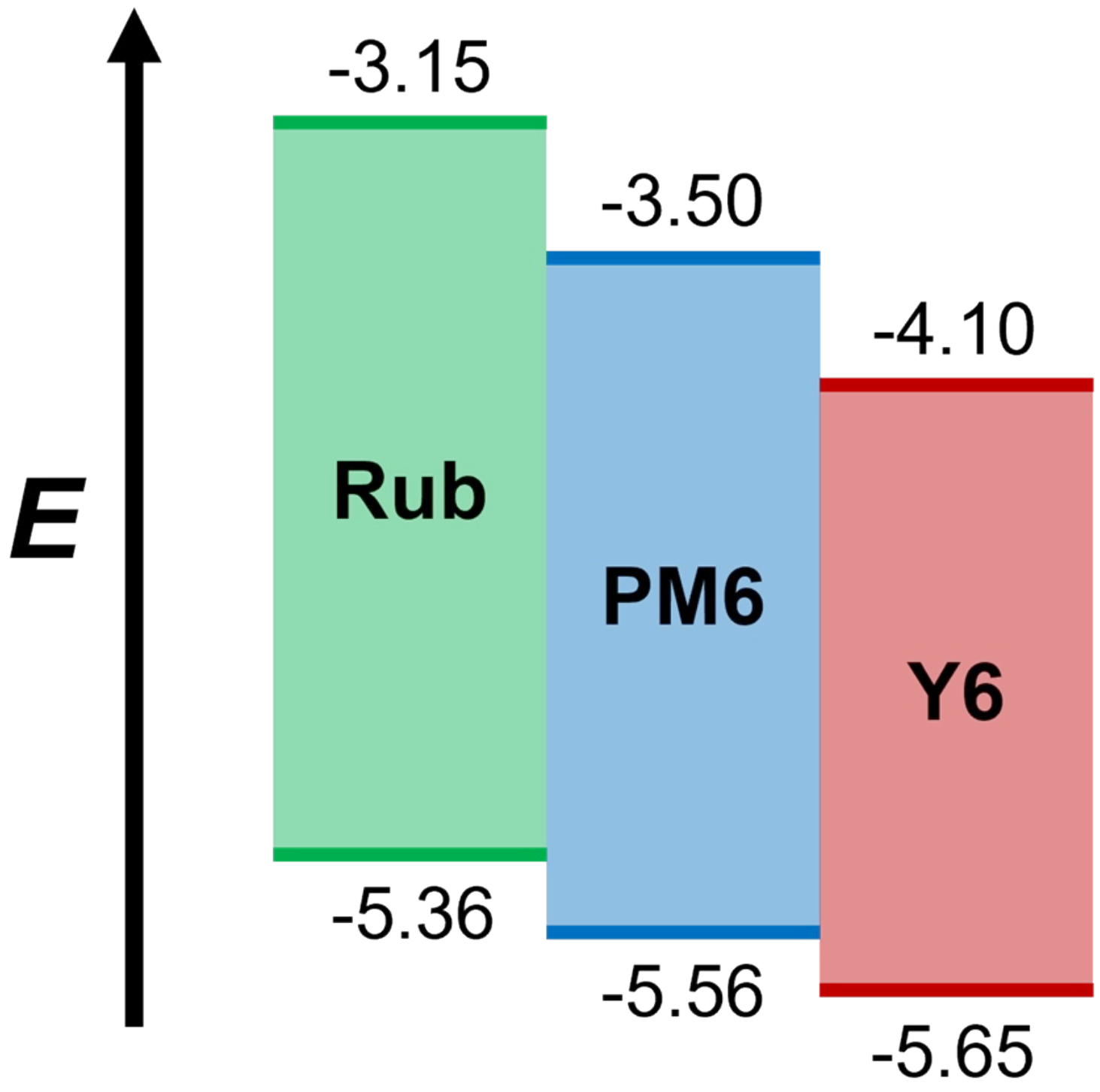

Figure S4. The estimated HOMO and LUMO values for rubrene[10], PM6, and Y6[11], as measured by cyclic voltammetry in the literature.

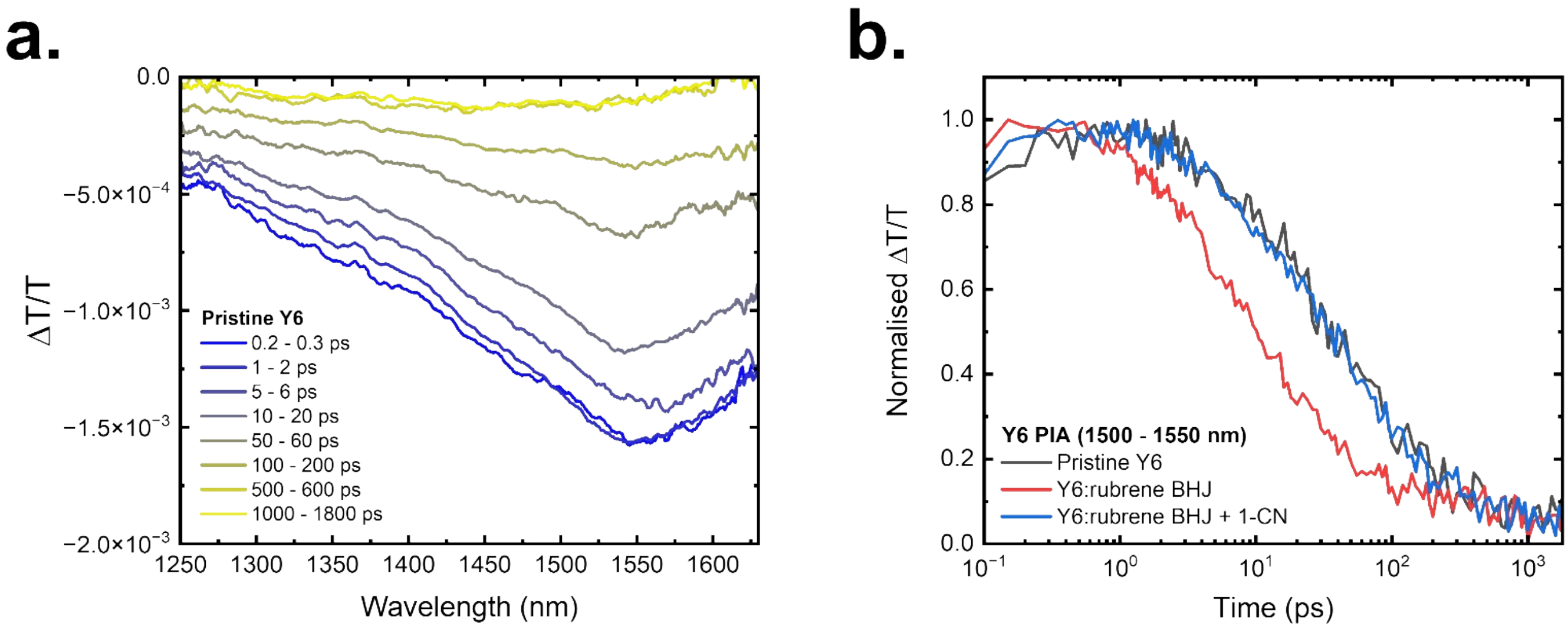

Figure S5. a. The TA spectra of a pristine Y6 film measured in the infrared spectral region. **b.** The TA kinetics of the Y6 hole polaron PIA (1500 – 1550 nm) of a pristine Y6 film, a Y6:rubrene BHJ film (4:1) without solvent additives, and a Y6:rubrene (4:1) BHJ film with 0.5 v/v% of 1-chloronapthalene (1-CN) solvent additive. All measurements were performed with an excitation wavelength of 800 nm and a fluence of 2.1 μJ/cm^2.

Interpretation of the Y6:rubrene BHJ TA spectra

The TA kinetic of the Y6:rubrene (4:1) BHJ film without any solvent additive (Fig. S5b), we observe a clear reduction in the lifetime of the Y6 hole polaron PIA at 1550 nm, which we attribute to hole transfer from Y6 to rubrene. However, upon the addition of 0.5 v/v% 1-CN solvent additive to the precursor solution, we find that there is no clear quenching of the Y6 hole polaron lifetime in the BHJ with rubrene. One possible explanation is that the addition of 1-CN drives the phase separation of Y6 and rubrene, which greatly reduces the donor:acceptor interfacial area and thus impedes the hole transfer process. Finally, by 1 – 1.8 ns in the pristine Y6 film (Fig. S5a), we observe that only Y6 T_1 states with a PIA at 1450 nm remain.

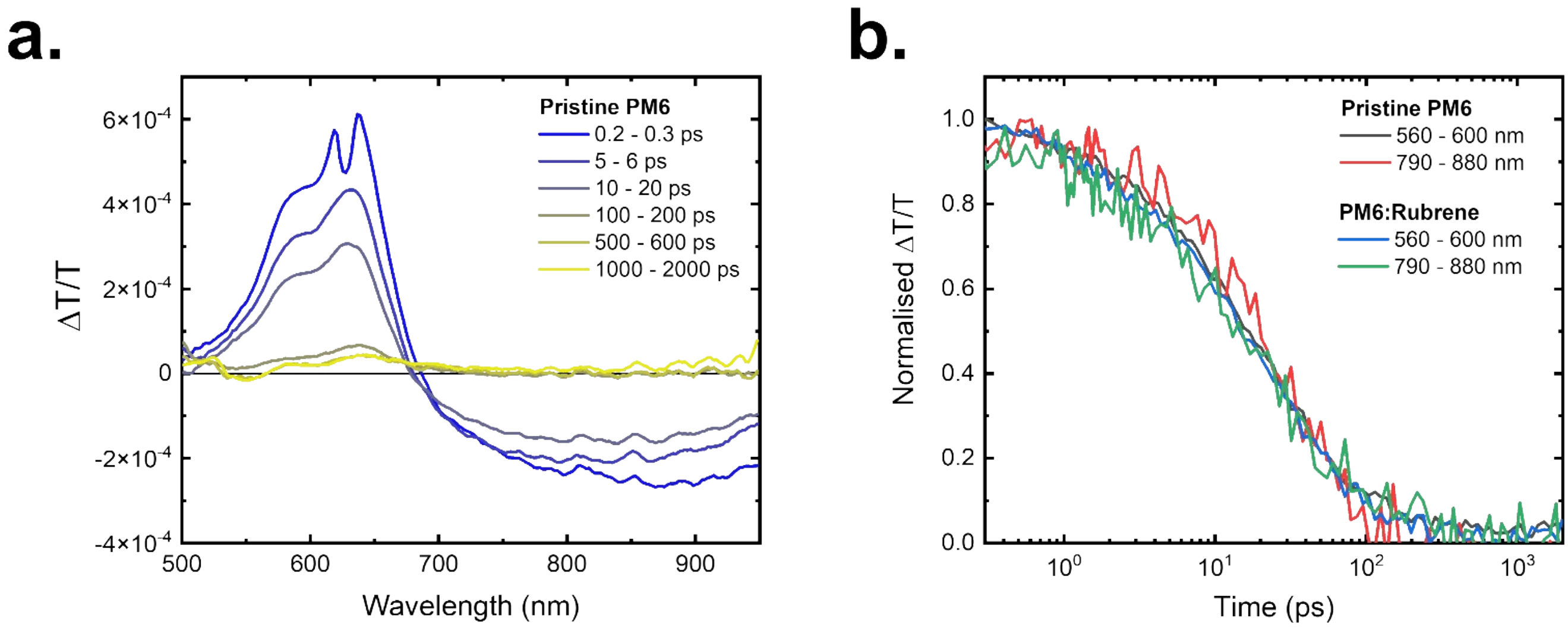

Figure S6. a. The TA spectra of a pristine PM6 film measured in the visible spectral region. **b.** The TA kinetics of the PM6 GSB (560 – 600 nm) and S_1 PIA (790 – 880 nm) of a pristine PM6 film and a PM6:rubrene BHJ film (4:1) without solvent additives. All TA measurements were performed with an excitation wavelength of 610 nm and a fluence of 1.0 μJ/cm^2.

Interpretation of the PM6:rubrene BHJ TA spectra

The TA kinetic of the PM6:rubrene (4:1) BHJ film without any solvent additive (Fig. S6b), we observe no change in the excited state lifetime across the spectrum when compared to a pristine PM6 film. Thus, based on the absence of quenching of PM6 S_1 states and lack of long-lived excited states, we conclude that there is not significant hole transfer from PM6 to rubrene. However, it is not clear whether this is due to the energetic driving force for hole transfer being too small, or the formation of an unfavourable phase-separated morphology with a small donor:acceptor interfacial area.

Calculations on the rubrene and PM6 FRET pair

The rate of Förster resonance energy transfer (FRET), k_{FRET}, between a donor and acceptor pair is given by:

$$k_{FRET} = \frac{1}{\tau}\left(\frac{R_0}{r}\right)^6 \#(1)$$

where τ is the fluorescence lifetime of the donor, r is the distance between the donor and acceptor and R_0 is the Förster radius. FRET will occur at a donor-acceptor separation in the range of 1 – 5 nm[1,2]. The Förster radius is related to the overlap integral between the donor emission and acceptor absorption, $J(\lambda)$, such that:

$$R_0^6 = \frac{9\ln(10)}{128\pi^5 N_A} \cdot \frac{\kappa^2 \Phi_D J(\lambda)}{n^4} \#(2)$$

where N_A is Avogadro's constant (6.022×10^{23} mol^{-1}), Φ_D is the fluorescence quantum yield of the donor and n is the refractive index. κ_2 is the dipole-dipole orientation factor and for polymers, a value of 0.476 is assumed, which considers a random orientation of dipoles in a film[3–5]. As introduced earlier, $J(\lambda)$ describes the degree of overlap between the normalised emission spectrum for the donor with the normalised absorption spectrum of the acceptor (by area):

$$J(\lambda) = \int f_D(\lambda)\varepsilon_A(\lambda)\lambda^4 \, d\lambda \#(3)$$

where f_D is the normalised emission spectrum of the donor, ε_A is the extinction coefficient of the acceptor[6]. $\varepsilon_A(\lambda)$ is equal to $\frac{A(\lambda)M_w}{d}$, where M_w is the molecular weight and d is the density[7]. A d of 1.2 kg L^{-1} is typical for polymer materials. Equation 2 can be further simplified to:

$$R_0 = 0.0211 \cdot \left(\frac{\kappa^2 \Phi_D J(\lambda)}{n^4}\right)^{\frac{1}{6}} \#(4)$$

The constant 0.0211 is used here if J is in units of L mol^{-1} cm^{-1} nm^{-1} and R_0 is given in nm. The constant is equal to $\left(\frac{9\ln(10)}{128\pi^5 N_A} \cdot 10^{17}\right)^{\frac{1}{6}}$. The parameters used for this calculation are summarised in Table S1 below.

Parameter	Value
M_w (kg mol^{-1})	100

d (kg L^{-1})	1.2
κ_2	0.476
Φ_D (%)	98
τ_D (s)	16.4×10^{-9} [8,9]
n	1.7
$J(\lambda)$ (L mol^{-1} cm^{-1} nm^{-1})	5.75×10^{15}
R_0 (nm)	5.2
k_{FRET} (r = 1 nm) (s^{-1})	1.73×10^{12}
τ_{FRET} (s)	5.8×10^{-13}

Table S1. The calculated FRET parameters for the rubrene (donor) and PM6 (acceptor) FRET pair.

Based on these calculations, we obtain R_0 = 5.2 nm, with a k_{FRET} at r = 1 nm of 1.73×10^{12} s^{-1} for the rubrene and PM6 FRET pair.

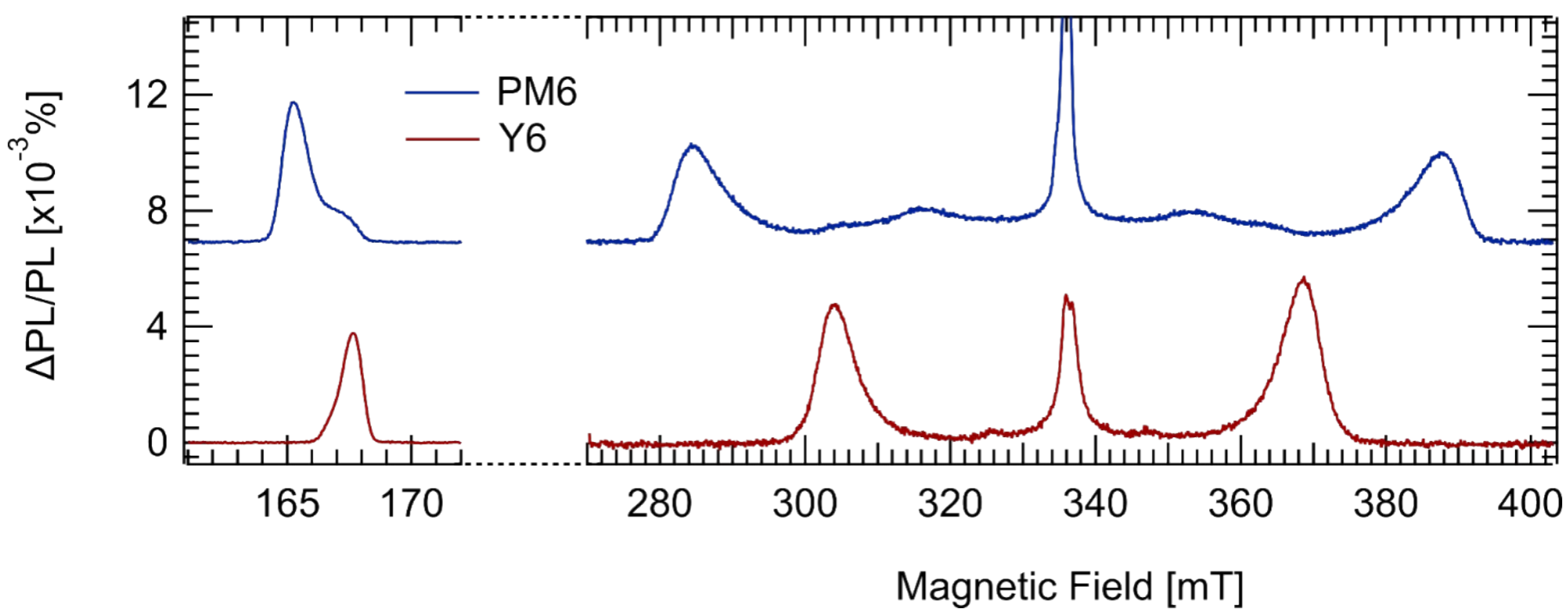

Figure S7. The PLDMR spectra for the pristine PM6 and Y6 films. Sample excitation was provided by a continuous wave laser at 532 nm, with a power of 80 mW, and detection at >561 nm. All measurements were carried out at 10 K.

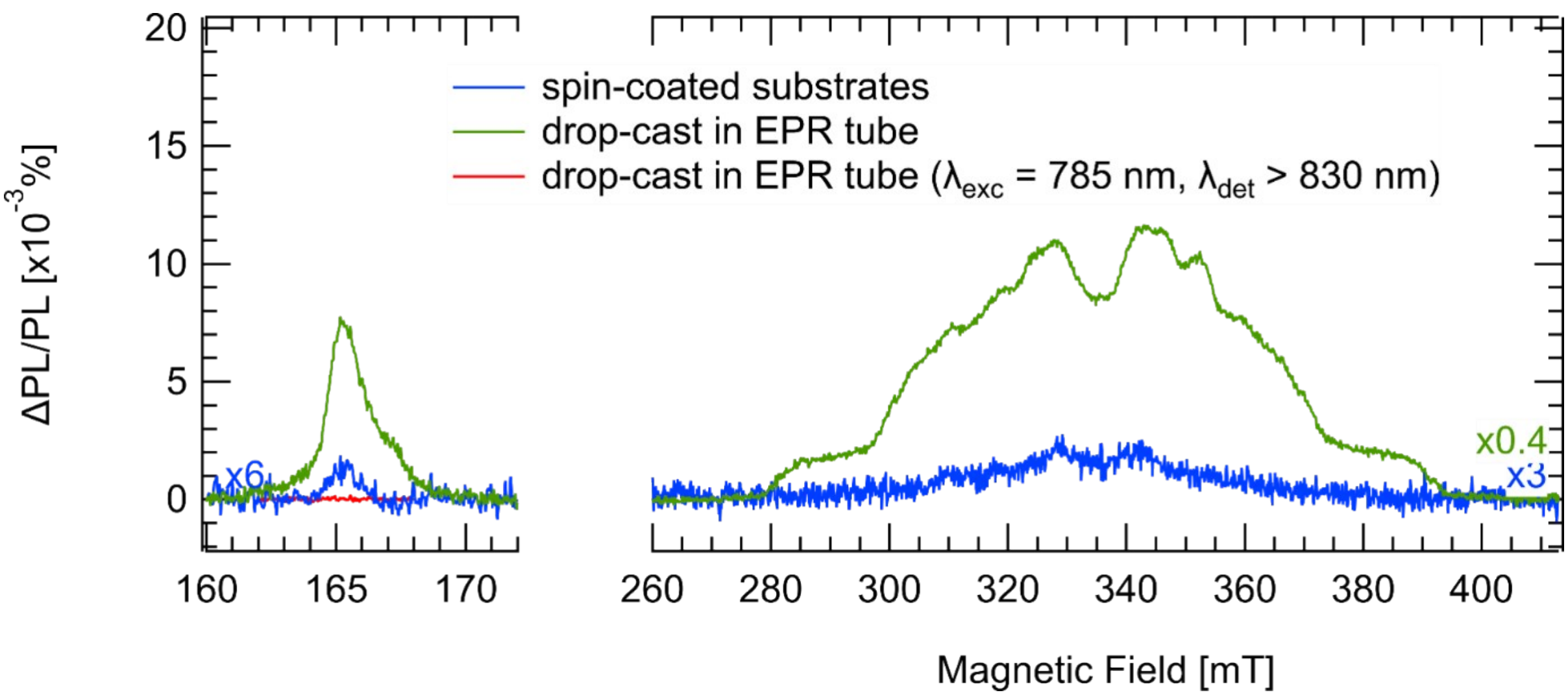

Figure S8. The PLDMR spectra of an evaporated pristine rubrene thin film and a more crystalline drop-cast rubrene film with a stronger PLDMR response. Sample excitation was provided by a continuous wave laser at 532 nm, with a power of 80 mW, and detection at >561 nm. The drop-cast rubrene film was also measured under identical conditions to the PM6:Y6:rubrene samples shown in Figure 2, using continuous wave laser excitation at 785 nm, with a power of 50 mW, and detection at >830 nm. This measurement was performed to rule out the presence of any PLDMR signal directly from rubrene under

this excitation condition, as confirmed by the measured baseline in the half field region. No spectral simulation of pristine rubrene was attempted, but the spectral width of ~115 mT corresponds to a ***D***-parameter of 1500 MHz. All measurements were carried out at 10 K.

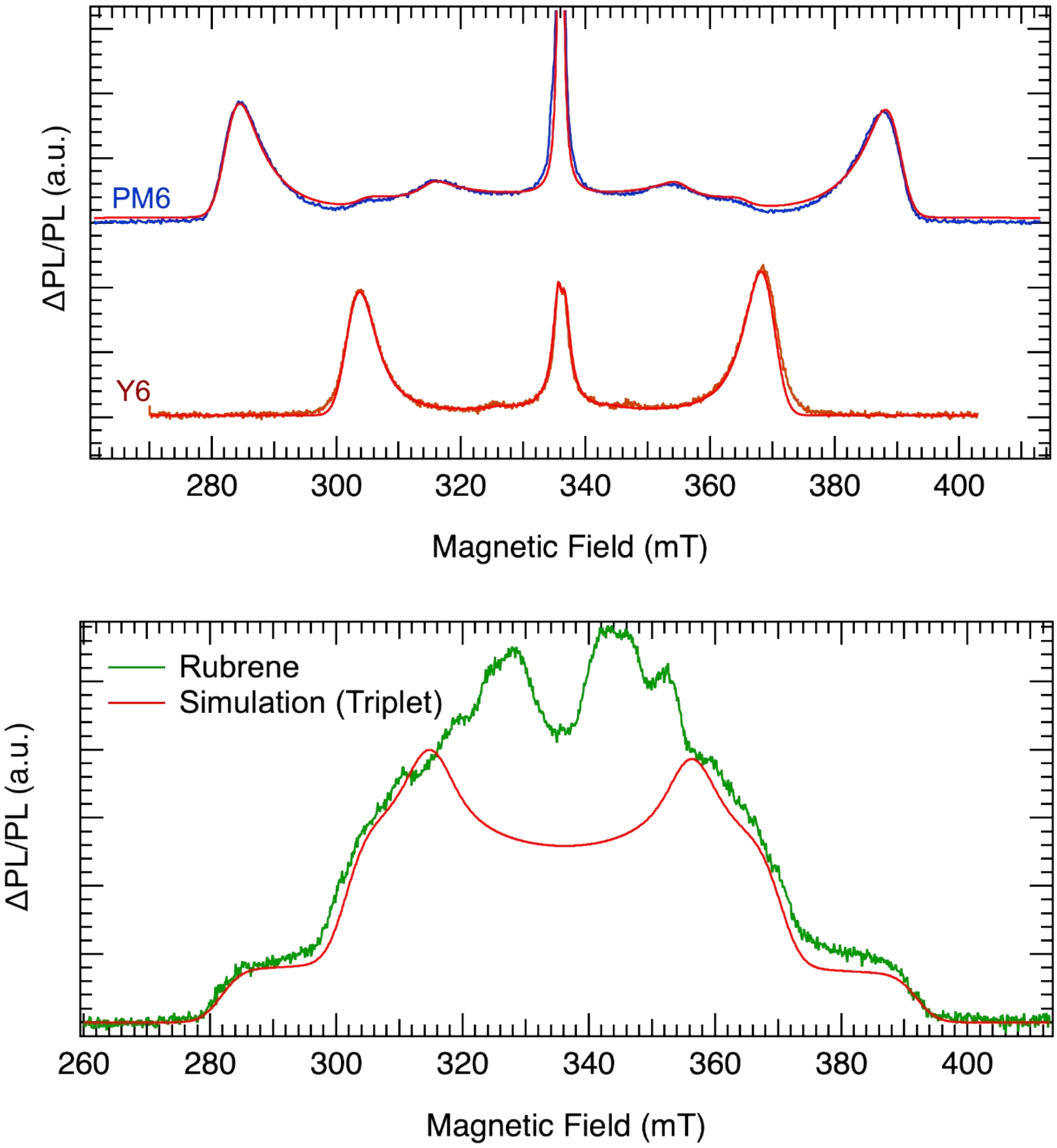

Figure S9. The PLDMR spectral simulations of the pristine PM6, Y6, and drop-cast rubrene samples. Note that the rubrene sample contains both triplet and quintet features, with the latter likely resulting from singlet fission. The triplet feature spans the whole spectral width from approximately 279 – 393 mT, whilst the quintet feature only occupies the centre of the spectrum. However, due to the complexity of simulating the quintet feature, we have chosen to only simulate the triplet feature, as this is the populated state in the PM6:Y6:rubrene films and is thus the signal most relevant to our current study.

	**Triplet**	**CT**

Sample	*D, E* (MHz)	$\lambda_\theta, \lambda_\varphi$	Lw (mT)	Weight	Lw (mT)	Weight
Y6	950, 150*<br>950, 150	11.0, 0<br>0.0, 0	4.0, 0<br>2.0, 0	0.62<br>0.11	1, 2.1<br>0, 1.3	0.27<br>-0.06
PM6	1510, 170<br>1510, 75<br>1510, 140	11.5, 0<br>-1.0, -2.5<br>3.5, 0	4.0, 0<br>0, 3.5<br>0, 4.0	0.40<br>0.11<br>0.33	0.3, 0.9	0.16
Rubrene	1550, 130	0.0, 0.0	6.0, 0	-	-	-

Table S2. The simulation values obtained from the PLDMR spectra of pristine PM6, Y6, and rubrene.

*ZFS ***E***-value not determinable due to high molecular ordering, determined by rotation measurements; Linewidth (Lw) in [Gaussian, Lorentzian]

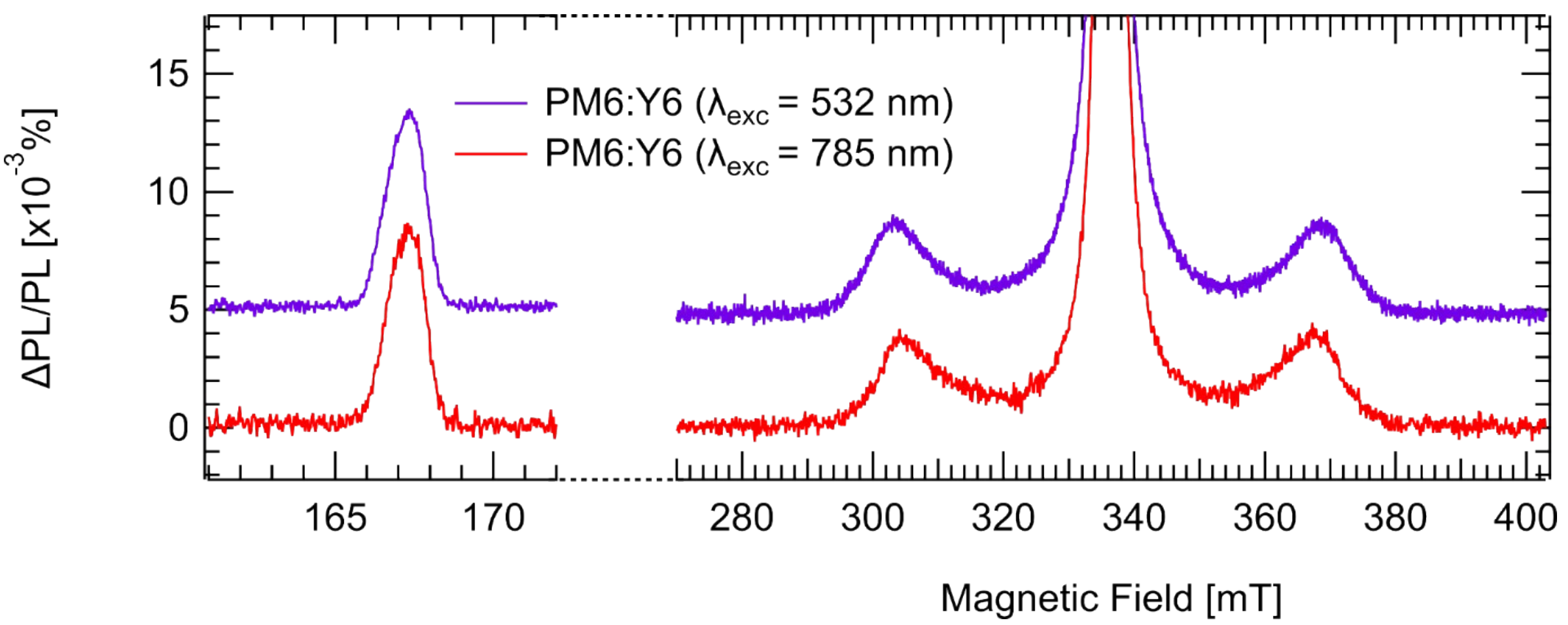

Figure S10. The PLDMR spectra of a pristine PM6:Y6 film with preferential excitation of PM6 and selective excitation of Y6. For the preferential excitation of PM6, a continuous wave laser at 532 nm

with a power of 80 mW was used (detection at >561 nm). For the selective excitation of Y6, a continuous wave laser at 785 nm with a power of 50 mW was used (detection at >830 nm). All measurements were carried out at 10 K.

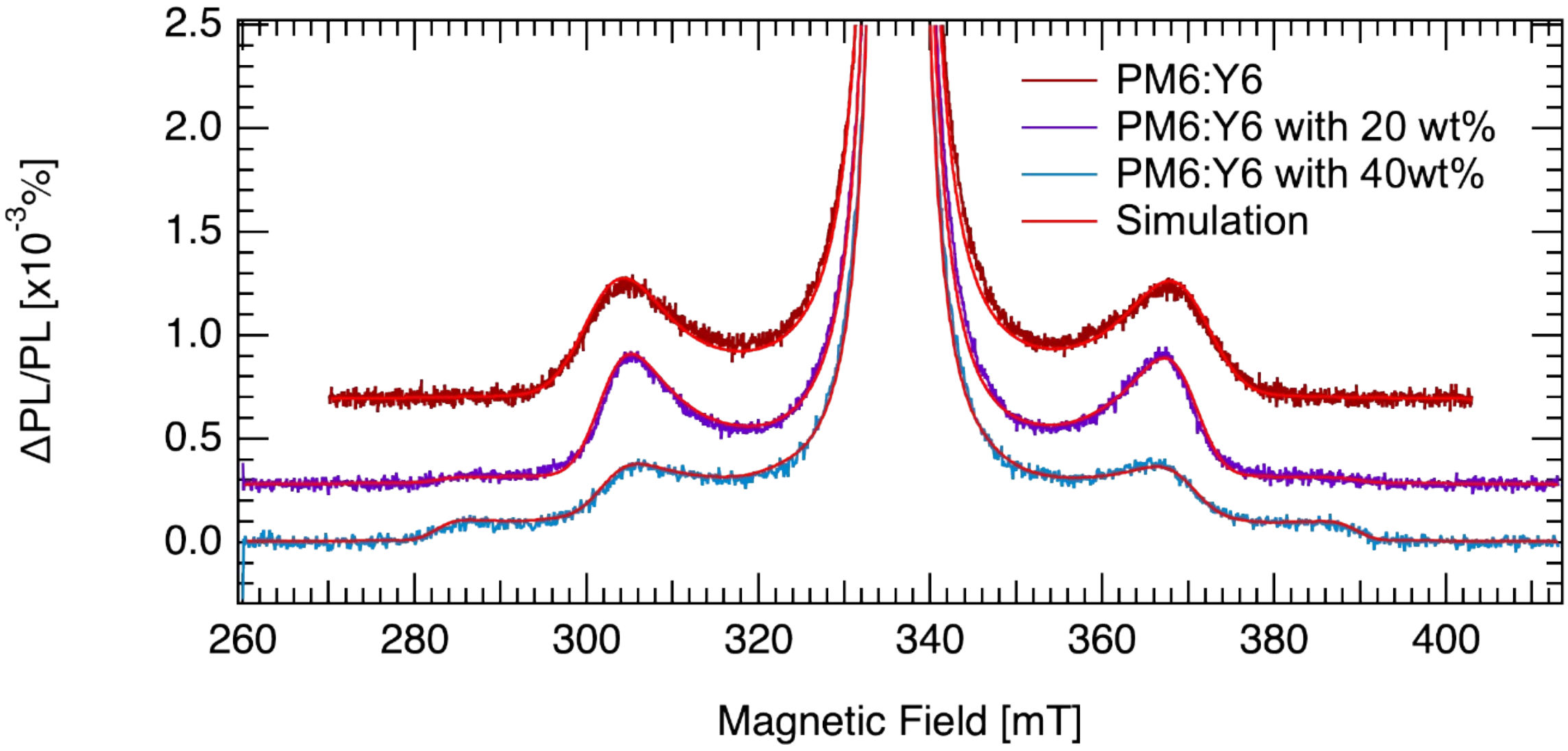

Figure S11. The PLDMR spectral simulations of the pristine PM6:Y6 and 20 and 40 wt% PM6:Y6:rubrene samples.

	**Triplet 1 (Y6)**				**Triplet 2 (rubrene)**			**CT state**	
**Sample**	***D*, *E* [MHz]**	**λ_{Θ}, λ_{φ}**	**L_w [mT]**	**Weight**	***D*, *E* [MHz]**	**Lw [mT]**	**Weight**	**Lw [mT]**	**Weight**
**Pristine PM6:Y6**	970, 220*	5.5, 0	7.8, 0	0.19	-	-	-	1.0, 2.1	0.81

**20 wt% rubrene**	960, 220*	5.5, 0	6.0, 0	0.11	1500, 180	4.0, 0	0.02	1.1, 2.2	0.87
**40 wt% rubrene**	960, 220*	2.7, 0	6.0, 0	0.09	1500, 180	4.0, 0	0.07	1.1, 1.9	0.84

Table S3. The simulation values obtained from the PLDMR spectra of pristine PM6:Y6, and PM6:Y6 films with 20 and 40 wt% rubrene.

*ZFS ***E***-value not determinable due to high molecular ordering, determined by rotation measurements of Y6; Linewidth (Lw) in [Gaussian, Lorentzian]

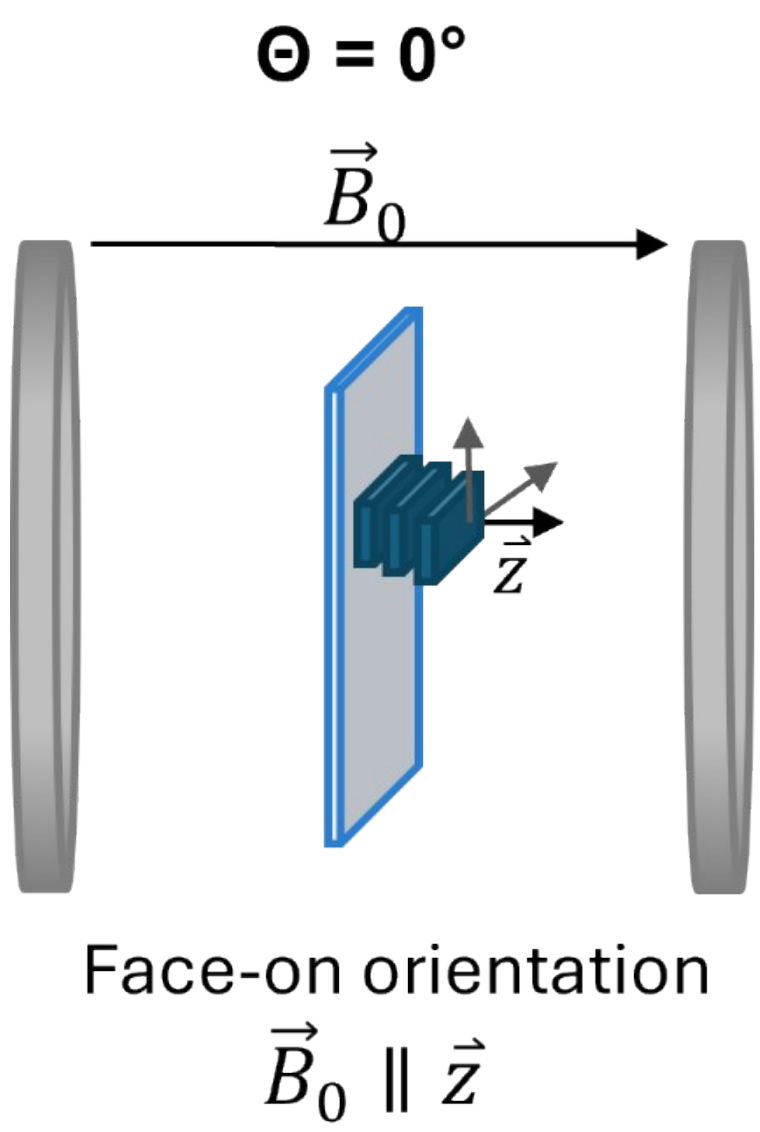

Figure S12. A schematic showing the orientation of the substrate to the external magnetic field in the PLDMR experiment. Note that the Y6 molecules preferentially orientate face-on to the substrate, resulting in the preferential ordering seen in the PLDMR measurement.

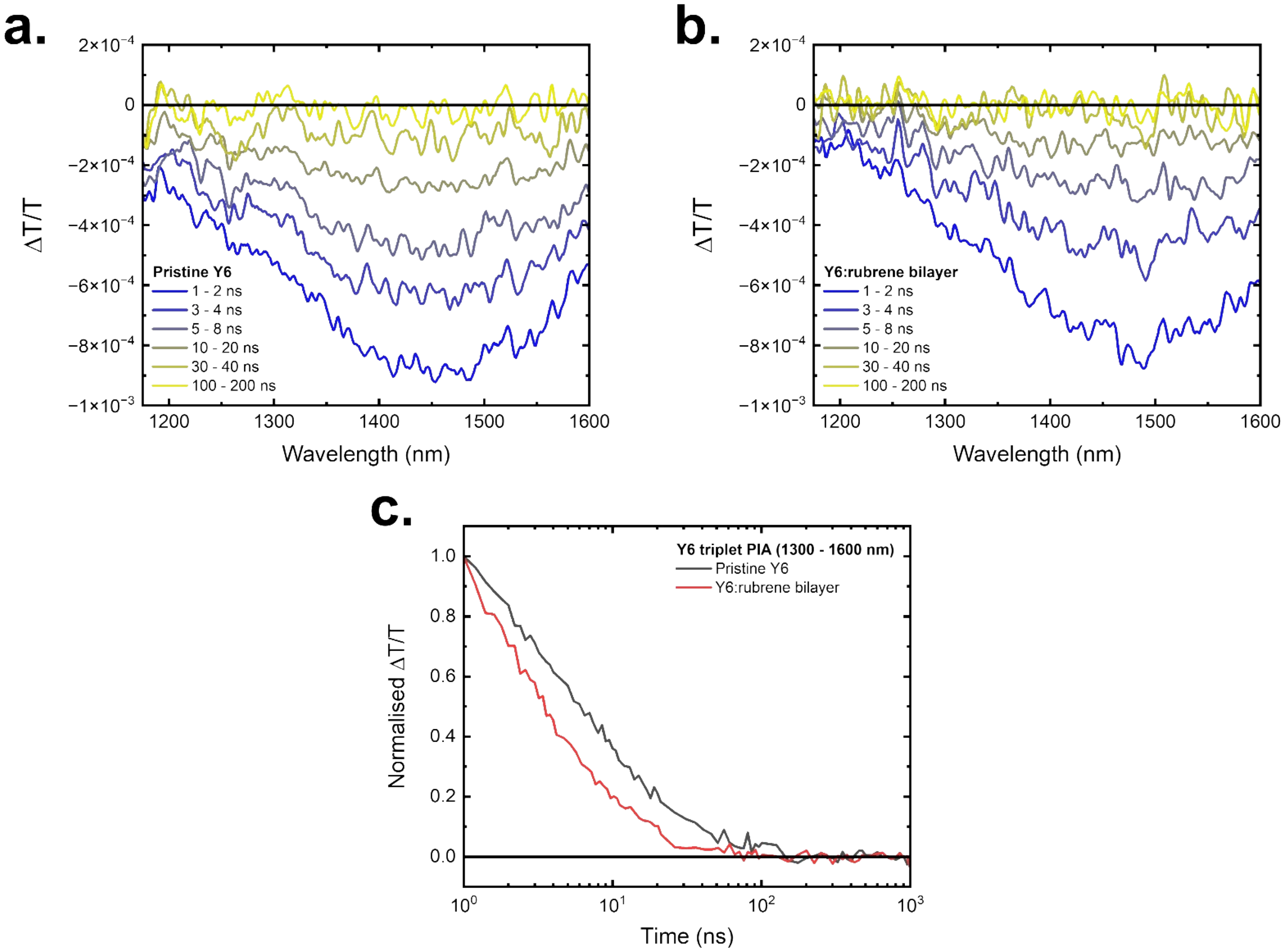

Figure S13. a. The nanosecond TA spectra of a pristine Y6 film measured in the infrared spectral region. The spectra were acquired with an excitation wavelength of 800 nm and a fluence of 23.1 $\mu J/cm^2$. **b.** The TA spectra of a Y6:rubrene bilayer film measured in the infrared spectral region. The spectra were acquired with an excitation wavelength of 800 nm and a fluence of 23.1 $\mu J/cm^2$. **c.** The TA kinetics taken from the Y6 T_1 PIA (1300 – 1600 nm) for the pristine Y6 and Y6:rubrene bilayer films.

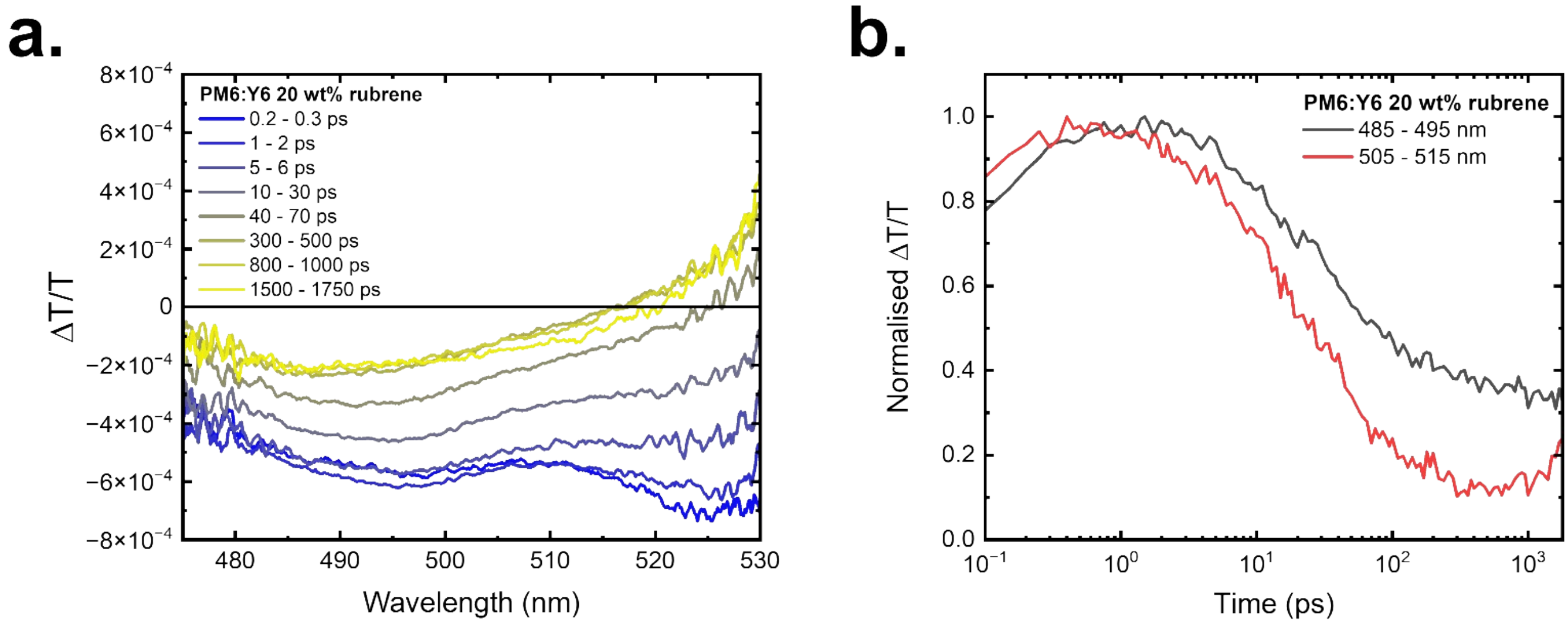

Figure S14. a. The TA spectra of a PM6:Y6 film with 20 wt% rubrene measured in the rubrene T_1 PIA region. The spectra were acquired with an excitation wavelength of 800 nm and a fluence of 5.1 µJ/cm^2. **b.** The TA kinetics taken from 485 – 495 nm and 505 – 515 nm, which show divergent behaviour. This underlines the complexity of the TA response for the PM6:Y6:rubrene sample in this spectral region.

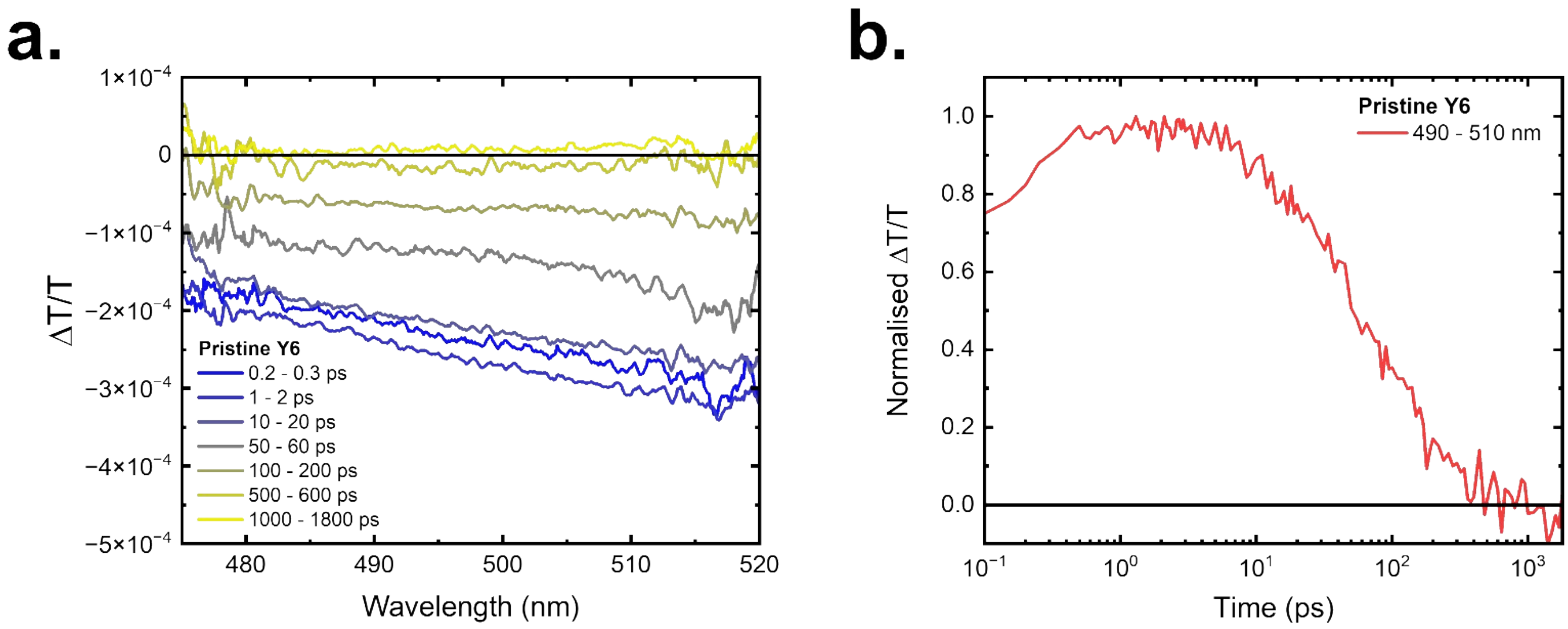

Figure S15. a. The TA spectra of a pristine Y6 film measured in the rubrene T_1 PIA region. The spectra were acquired with an excitation wavelength of 800 nm and a fluence of 2.9 μJ/cm^2. **b.** The TA kinetic taken from the PIA between 490 – 510 nm.

Interpretation of the TA spectra in the 475 – 530 nm region

The TA of PM6:Y6 with 20 wt% rubrene (Fig. S14a) shows a complex form in the 475 – 530 nm spectral region, consisting of several overlapping features that complicates the interpretation. At 0.2 – 0.3 ps following preferential excitation of Y6 at 800 nm, the spectrum appears to be a combination of the weak PIA band of Y6 seen in Fig. S15a, with a dip at around 510 nm. As the kinetic of the Y6 PIA in Fig. S15b matches that of the hole polaron PIA at 1550 nm (Fig. S5b), we consider that the visible-region PIA represents another weak optically allowed transition of the hole polaron, constituting the positively charged part part of the bound polaron pairs (PPs) generated in pristine Y6 domains (note that the Y6 electron polaron has a PIA at 780 nm)[12–14]. The origin of the dip in the PIA at 510 nm is not immediately clear but may be related to a weak vibronic shoulder of the PM6 GSB (Fig. S6) from the small amount of hole transfer that has already taken place by 0.2 ps[15].

As hole transfer progresses, the PIA in this region decreases in intensity, likely due to the quenching of the PIA band associated with hole polarons on Y6. Once hole transfer is completed after a few hundred picoseconds, the spectrum shows a clear PIA at 490 nm, with the edge of the PM6 GSB signal also visible around 530 nm. Finally, beyond 1 ns, we also see a slight growth in the edge of the main PIA at 510 nm. The presence of a PIA band at around 490 – 510 nm is consistent with the population of the rubrene T_1 state, which is well known to possess a PIA in this spectral region[16–18]. However, due to the complexity of the dynamics, as well as at a least a partial overlap of the rubrene T_1 PIA with the PM6 GSB, we instead focus our analysis on the infrared spectral region of these samples, where the dynamics of T_1 states on Y6 can be more clearly resolved.

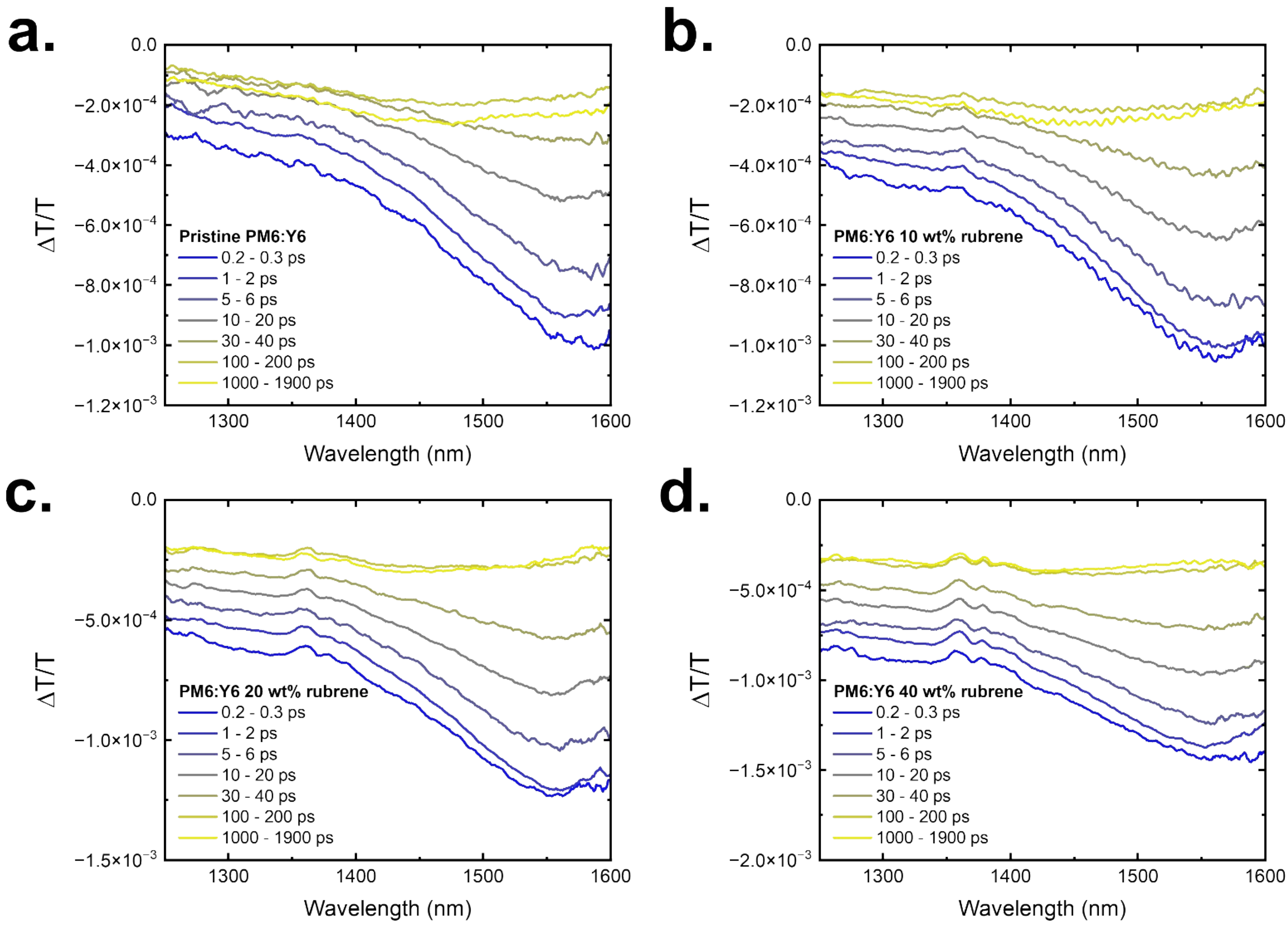

Figure S16. a. The TA spectra of a pristine PM6:Y6 film measured in the infrared spectral region. An excitation fluence of 2.8 μJ/cm² was used. **b.** The TA spectra of a PM6:Y6 film with 10 wt% rubrene measured in the infrared spectral region. An excitation fluence of 3.2 μJ/cm² was used. **c.** The TA spectra of a PM6:Y6 film with 20 wt% rubrene measured in the infrared spectral region. An excitation fluence of 3.6 μJ/cm² was used. **b.** The TA spectra of a PM6:Y6 film with 40 wt% rubrene measured in the infrared spectral region. An excitation fluence of 3.8 μJ/cm² was used. All measurements were performed with an excitation wavelength of 800 nm.

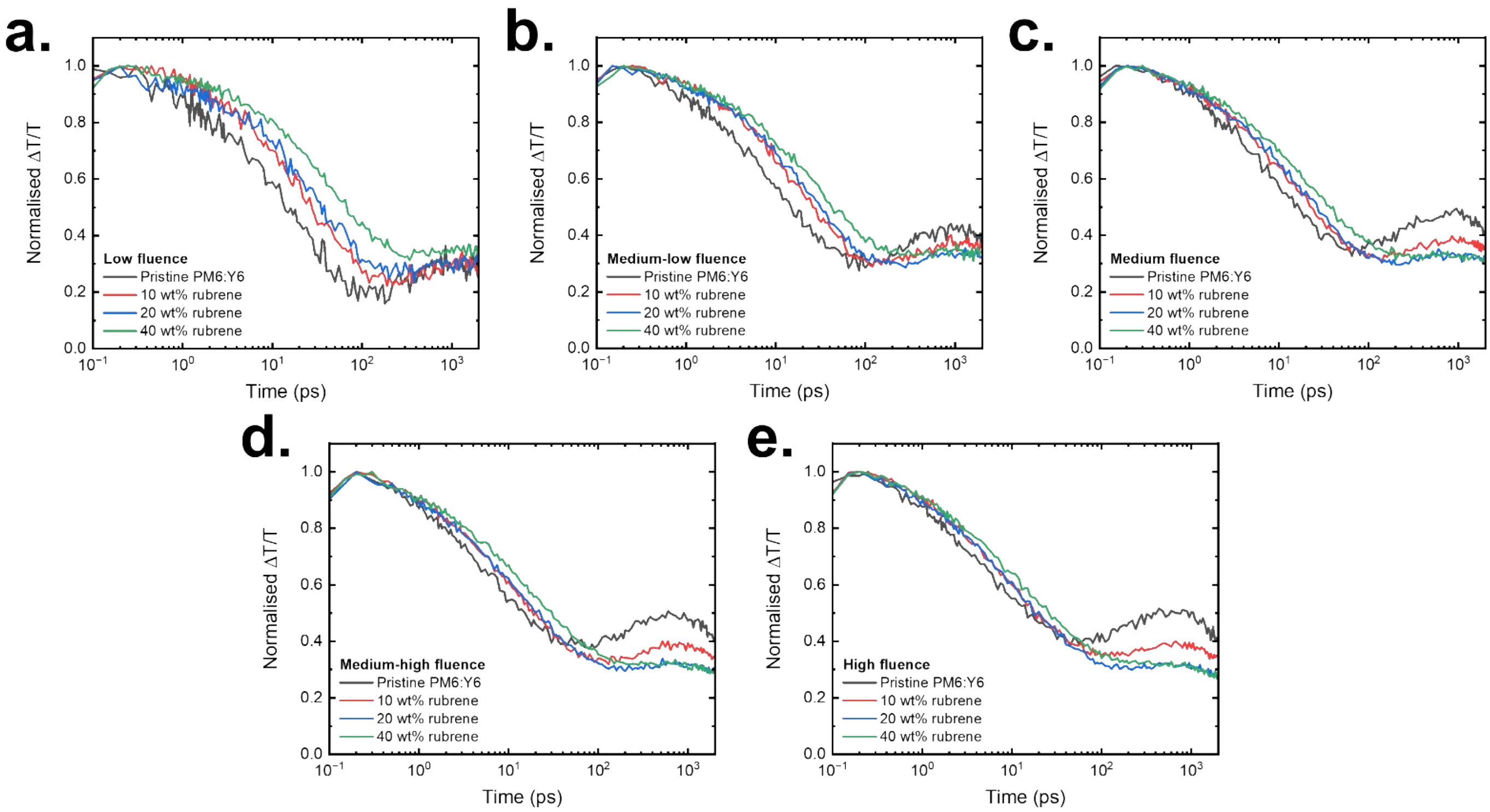

Figure S17. a. The normalised kinetics taken from the Y6 T_1 PIA region (1425 – 1475 nm) of a pristine PM6:Y6 film and PM6:Y6 films with 10, 20, and 40 wt% rubrene doping. The lowest fluence measurement (1.4 to 1.9 μJ/cm^2) is plotted for each film. **b.** The normalised kinetics taken from the Y6 T_1 PIA region (1425 – 1475 nm) of a pristine PM6:Y6 film and PM6:Y6 films with 10, 20, and 40 wt% rubrene doping. The medium-low fluence measurement (2.8 to 3.8 μJ/cm^2) is plotted for each film. **c.** The normalised kinetics taken from the Y6 T_1 PIA region (1425 – 1475 nm) of a pristine PM6:Y6 film and PM6:Y6 films with 10, 20, and 40 wt% rubrene doping. The middle fluence measurement (4.2 to 5.7 μJ/cm^2) is plotted for each film. **d.** The normalised kinetics taken from the Y6 T_1 PIA region (1425 – 1475 nm) of a pristine PM6:Y6 film and PM6:Y6 films with 10, 20, and 40 wt% rubrene doping. The medium-high fluence measurement (5.6 to 7.6 μJ/cm^2) is plotted for each film. **e.** The normalised kinetics taken from the Y6 T_1 PIA region (1425 – 1475 nm) of a pristine PM6:Y6 film and PM6:Y6 films with 10, 20, and 40 wt% rubrene doping. The highest fluence measurement (7.0 to 9.5 μJ/cm^2) is plotted for each film. All measurements were performed with an excitation wavelength of 800 nm.

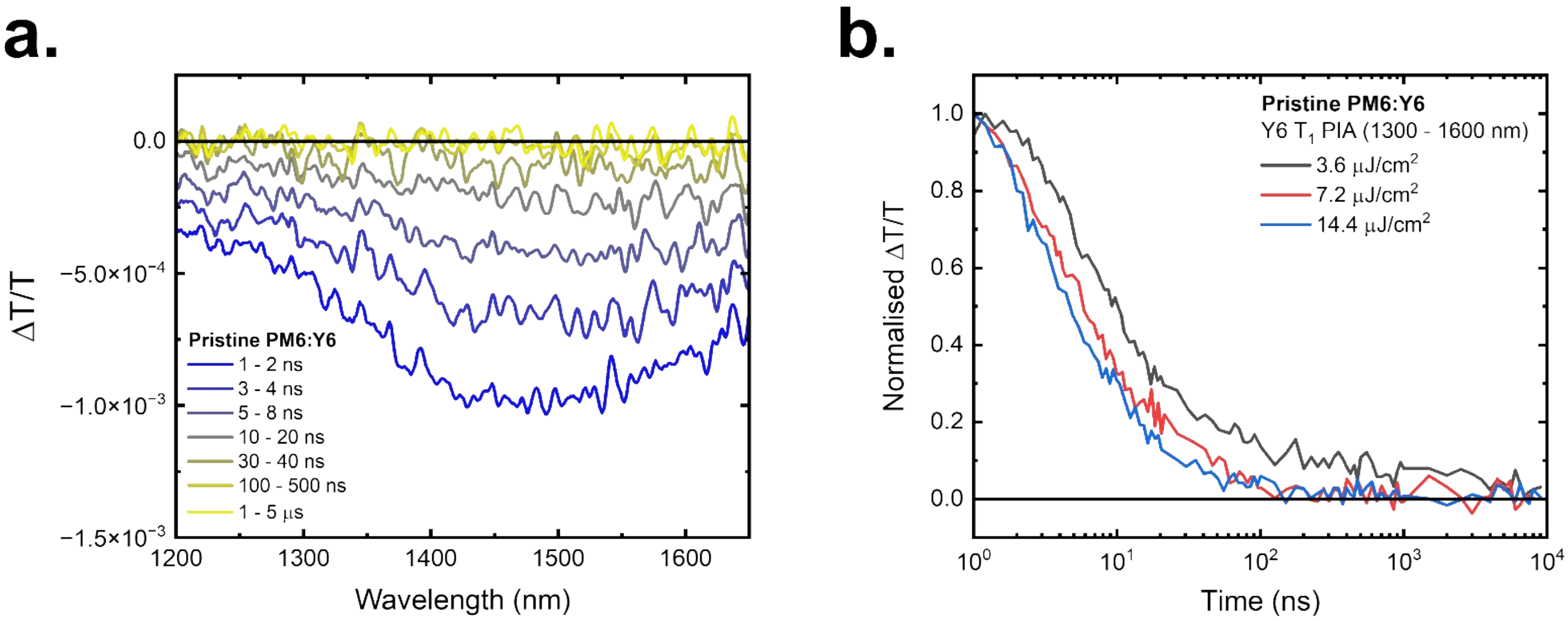

Figure S18. a. The nanosecond TA spectra of a pristine PM6:Y6 film measured in the infrared spectral region. The spectra were acquired with an excitation wavelength of 800 nm and a fluence of 14.4 μJ/cm^2. **b.** The TA kinetics taken over a fluence series from the Y6 T_1 PIA (1300 – 1600 nm) for the pristine PM6:Y6 film.

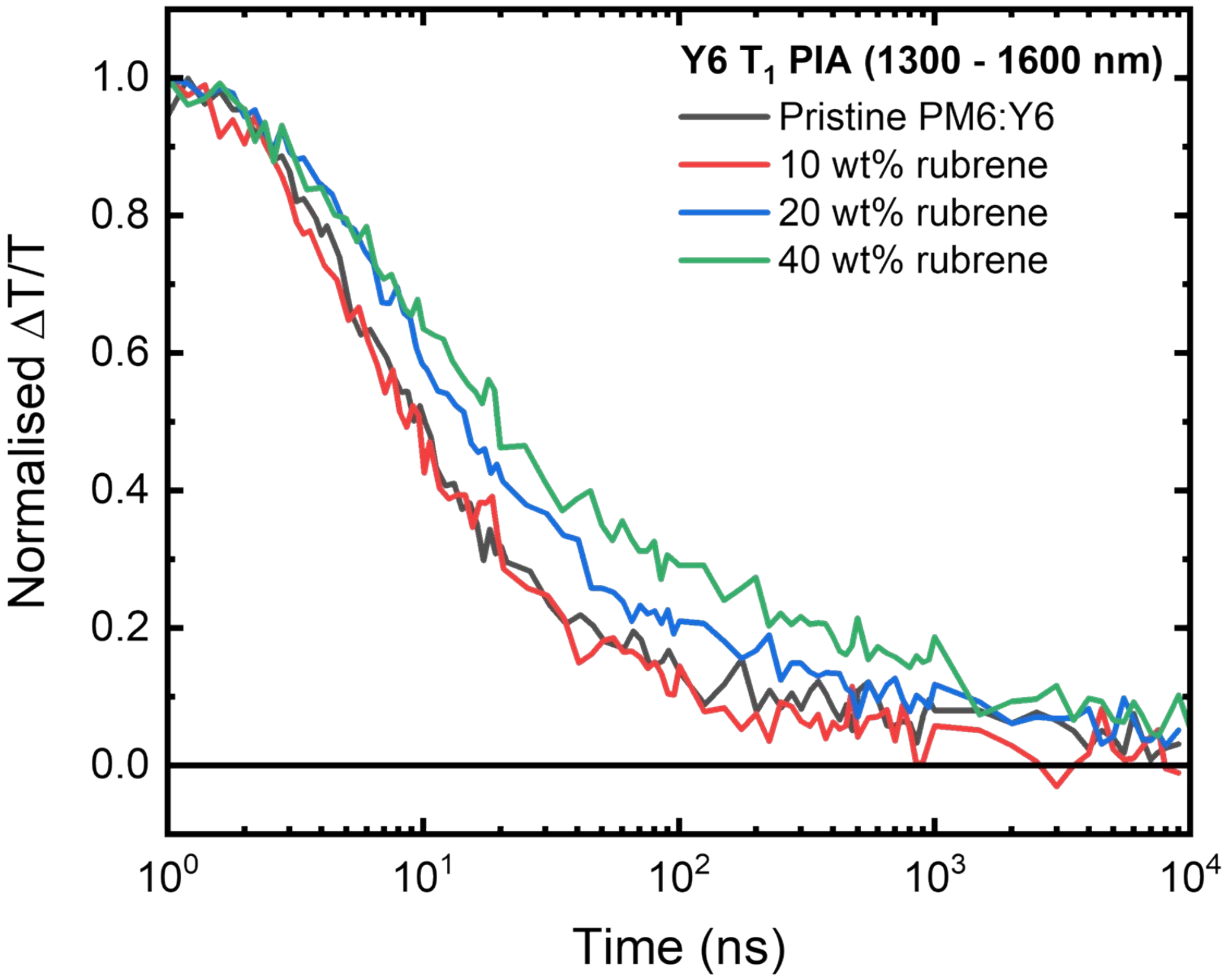

Figure S19. The nanosecond TA kinetics from the Y6 T_1 region (1300 – 1600 nm) of a pristine PM6:Y6 film, and films with 10, 20, and 40 wt% rubrene measured in the infrared spectral region. A fluence of 3.6 μJ/cm^2 was used for the pristine PM6:Y6 film, 4.2 μJ/cm^2 was used for the 10 wt% rubrene film, 4.8 μJ/cm^2 was used for the 20 wt% rubrene film, and 5.1 μJ/cm^2 was used for the 40 wt% rubrene film film.

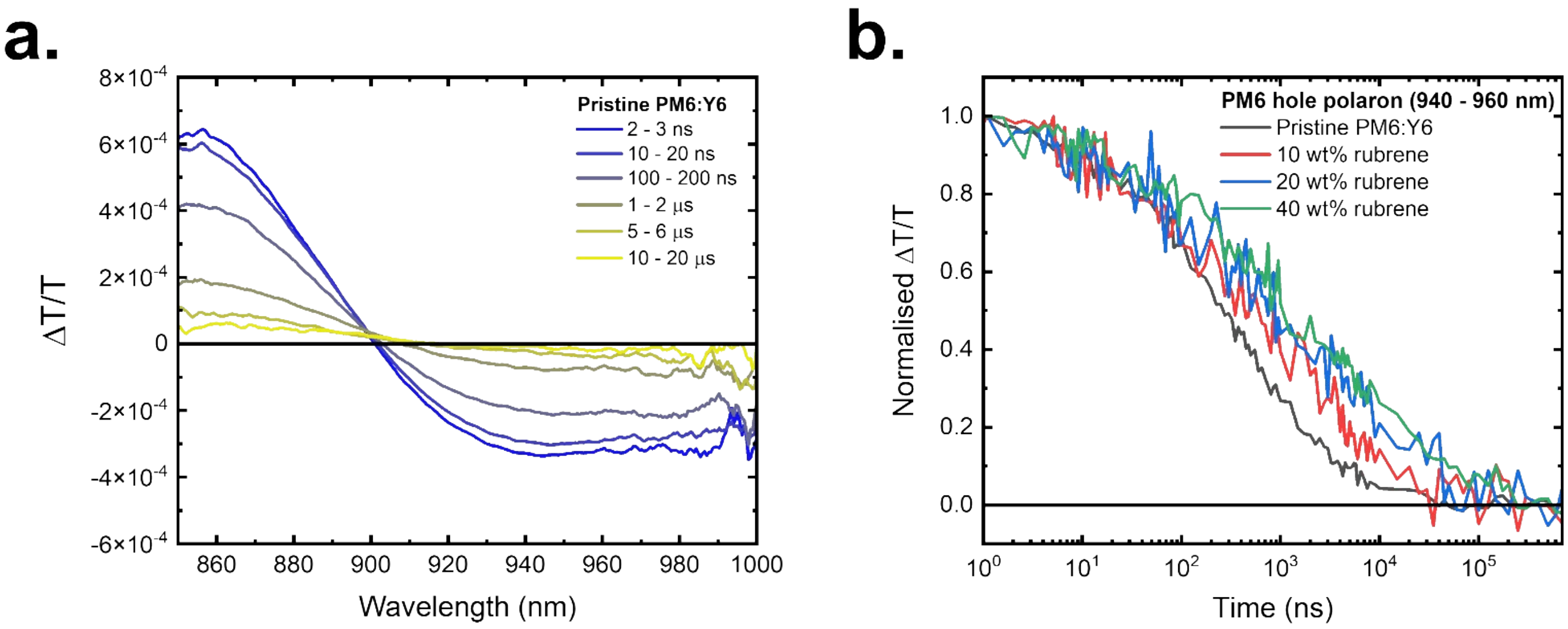

Figure S20. a. The nanosecond TA spectra of a pristine PM6:Y6 film measured in the near infrared spectral region (850 – 1000 nm). The spectra were acquired with an excitation wavelength of 532 nm and a fluence of 1.6 µJ/cm^2. **b.** The TA kinetics taken from the PM6 hole polaron PIA (940 – 960 nm) for the pristine PM6:Y6 film, and films with 10, 20, and 40 wt% rubrene.

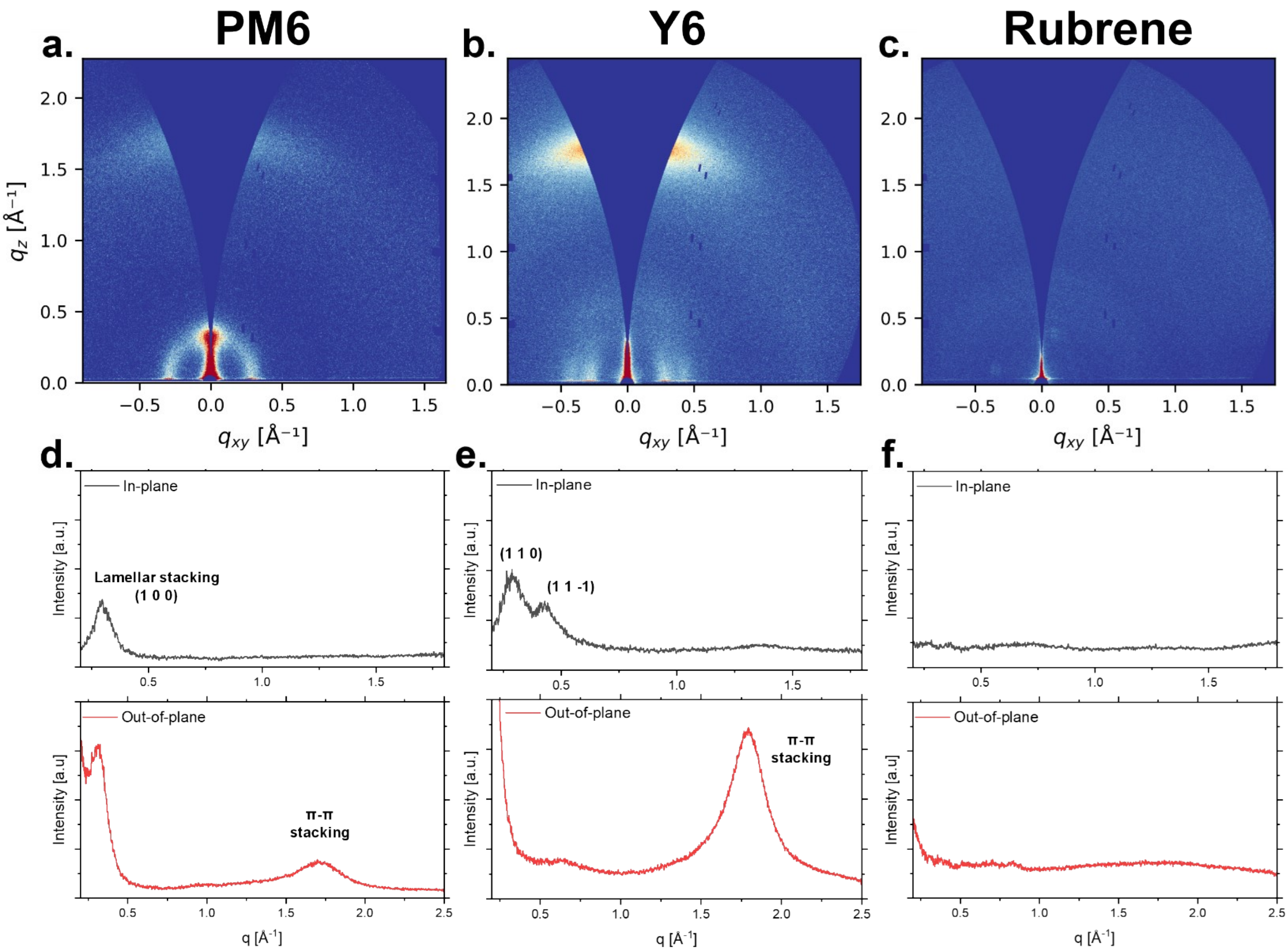

Figure S21. 2D GIWAXS patterns of pristine PM6, Y6 and rubrene films. The corresponding 1D azimuthally integrated intensity profiles in the in-plane and out-of-plane directions. The pristine PM6 film shows distinct scattering features at q = 0.294 $Å^{-1}$ (d = 21.4 Å) and q = 1.700 $Å^{-1}$ (d = 3.70 Å) consistent with scattering from lamellar stacking and π-π stacking respectively. The pristine Y6 film is characterised by two in-plane scattering features at q = 0.286 $Å^{-1}$ (d = 22.0 Å) and q = 0.432 $Å^{-1}$ (d = 14.5 Å) corresponding to the (1 1 0) and (1 1 -1) reflections as indexed elsewhere[19]. A distinct π-π stacking peak was also observed in the out-of-plane direction for the Y6 film at q = 1.782 $Å^{-1}$ (d = 3.53 Å). For the pristine rubrene film, no strong scattering features were observed indicating an amorphous molecular arrangement.

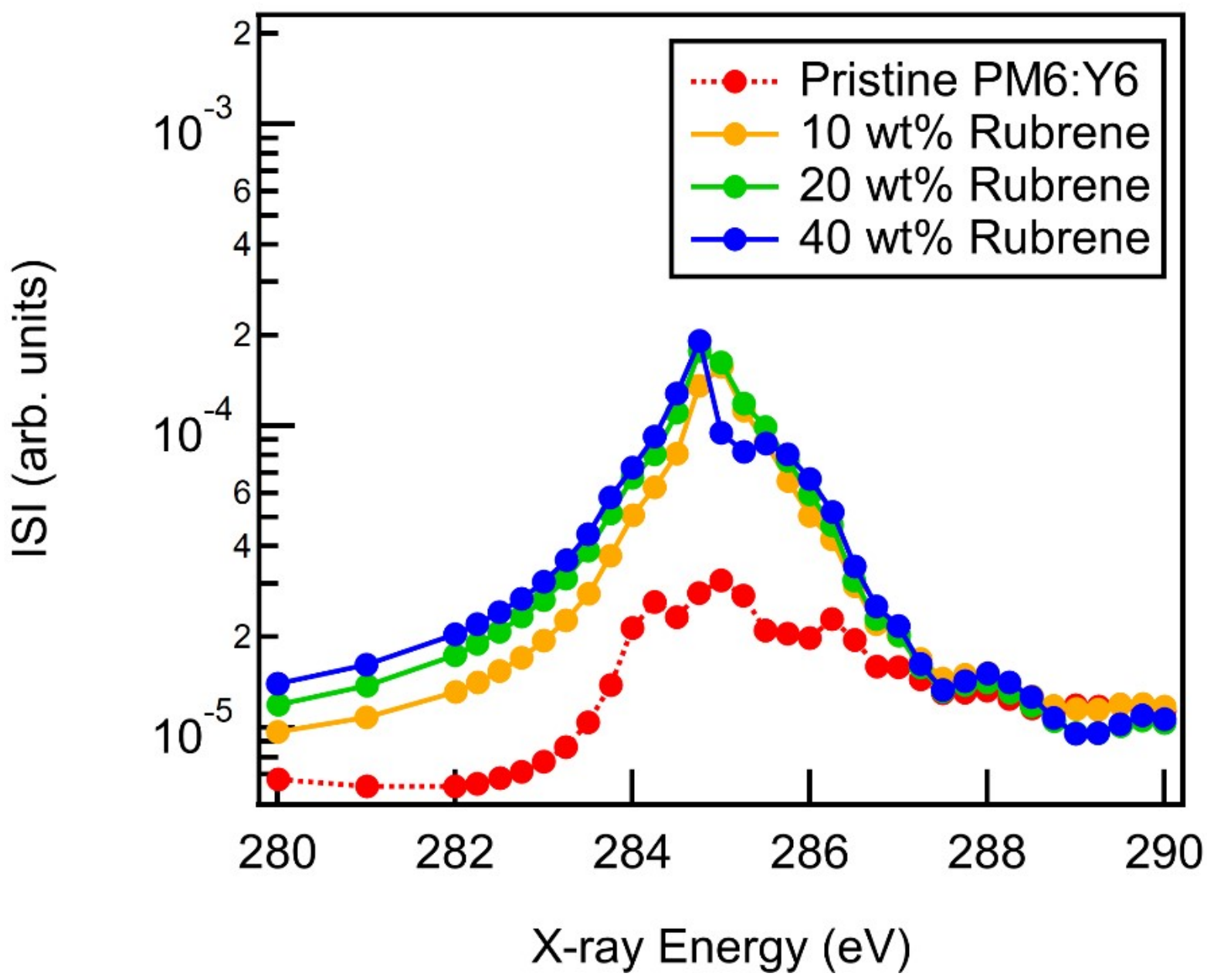

Figure S22. The energy dependence of the mass-roughness normalized R-SoXS integrated scattering intensity (ISI) around the carbon K-edge. The ISI follows typical material contrast functions, indicating that the observed scattering can be attributed to variations in material composition. The shift of the low-energy shoulder upon addition of rubrene likely indicates contributions to the scattering contrast from rubrene-containing (mixed) domains.

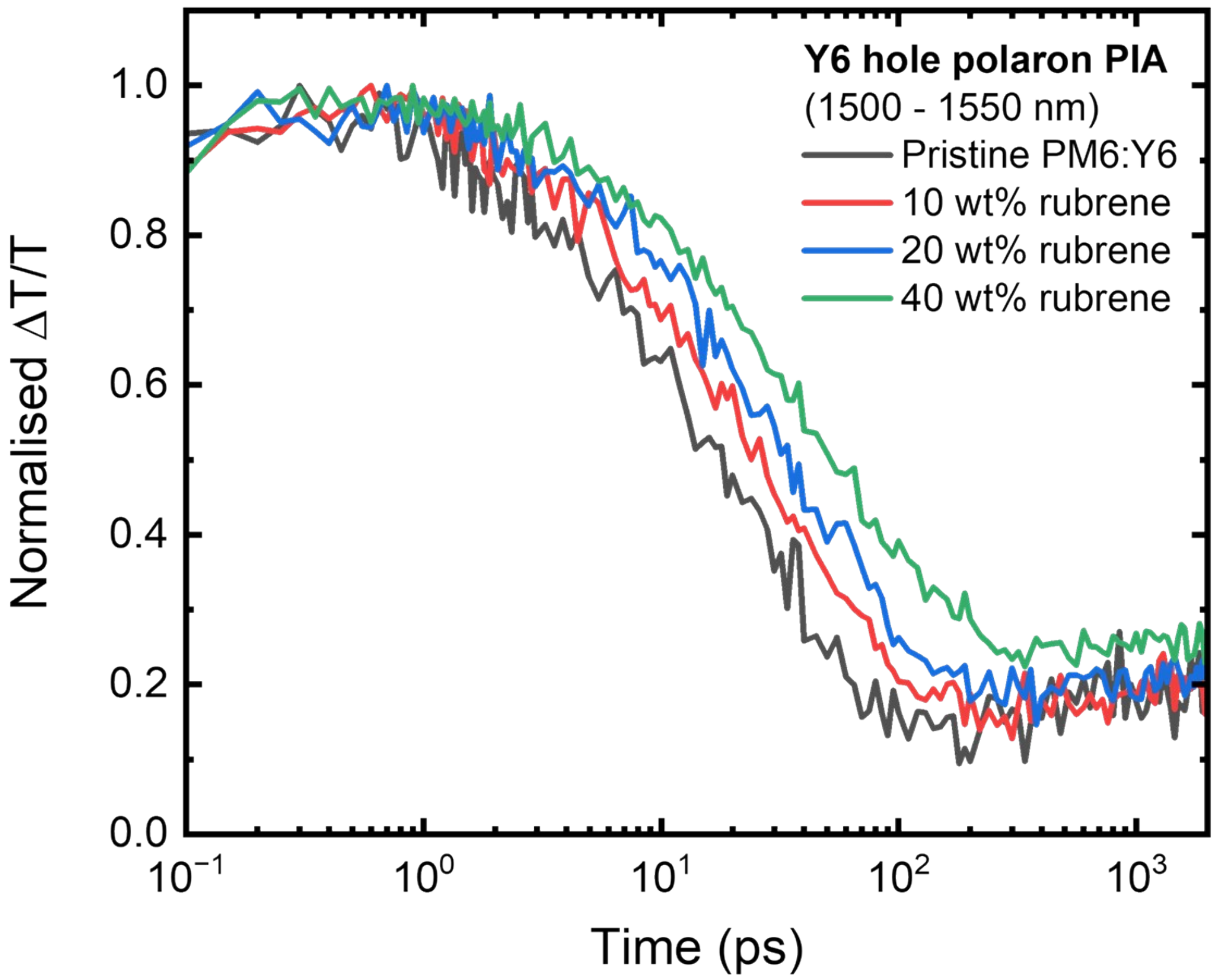

Figure S23. The normalised kinetics taken from the Y6 polaron PIA region (1500 – 1550 nm) of a pristine PM6:Y6 film and PM6:Y6 films with 10, 20, and 40 wt% rubrene doping. The lowest fluence measurement (1.4 to 1.9 μJ/cm²) is plotted for each film.

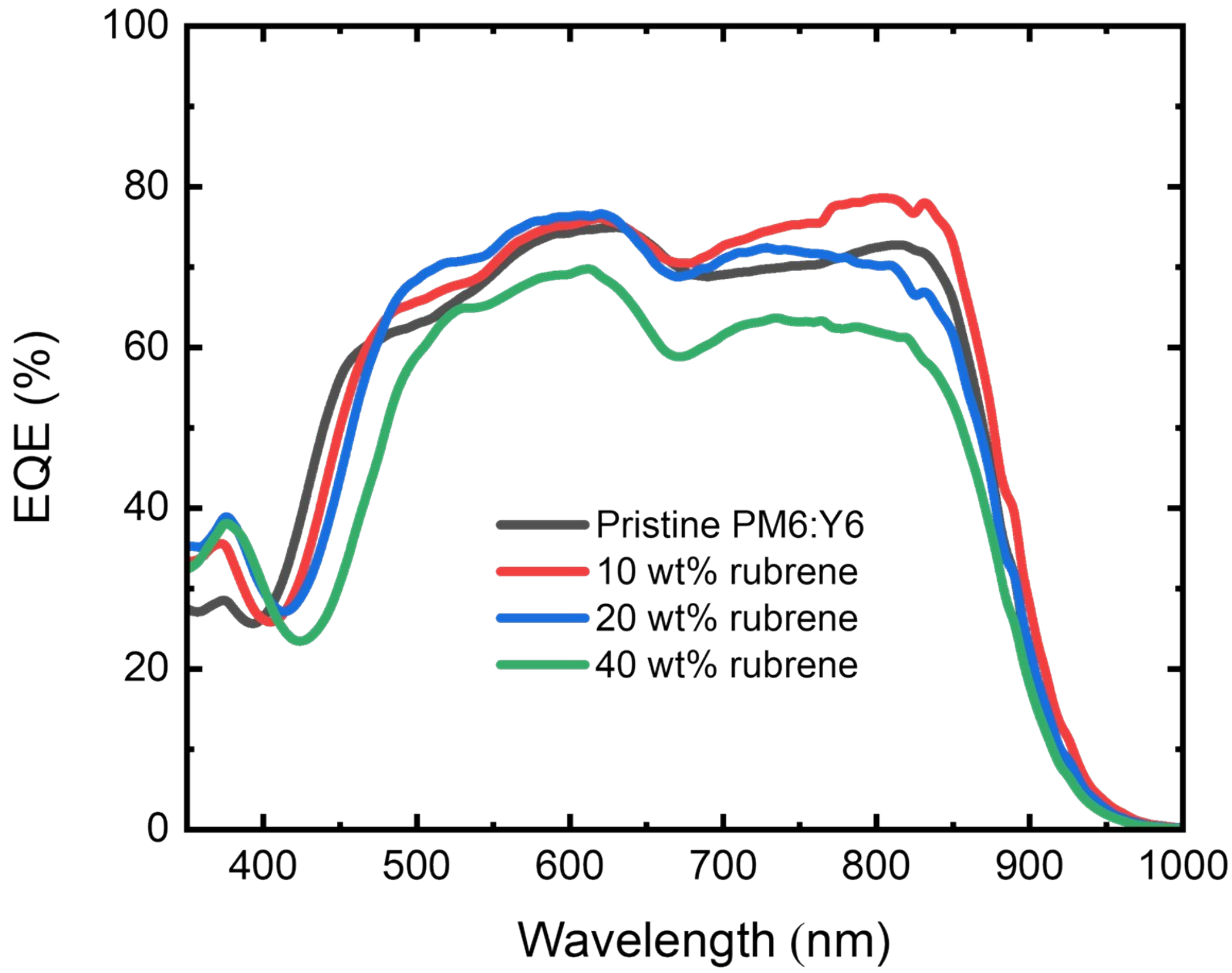

Figure S24. The EQE curves for the pristine PM6:Y6 OSC device, and PM6:Y6 devices with 10, 20, and 40 wt% rubrene doping.

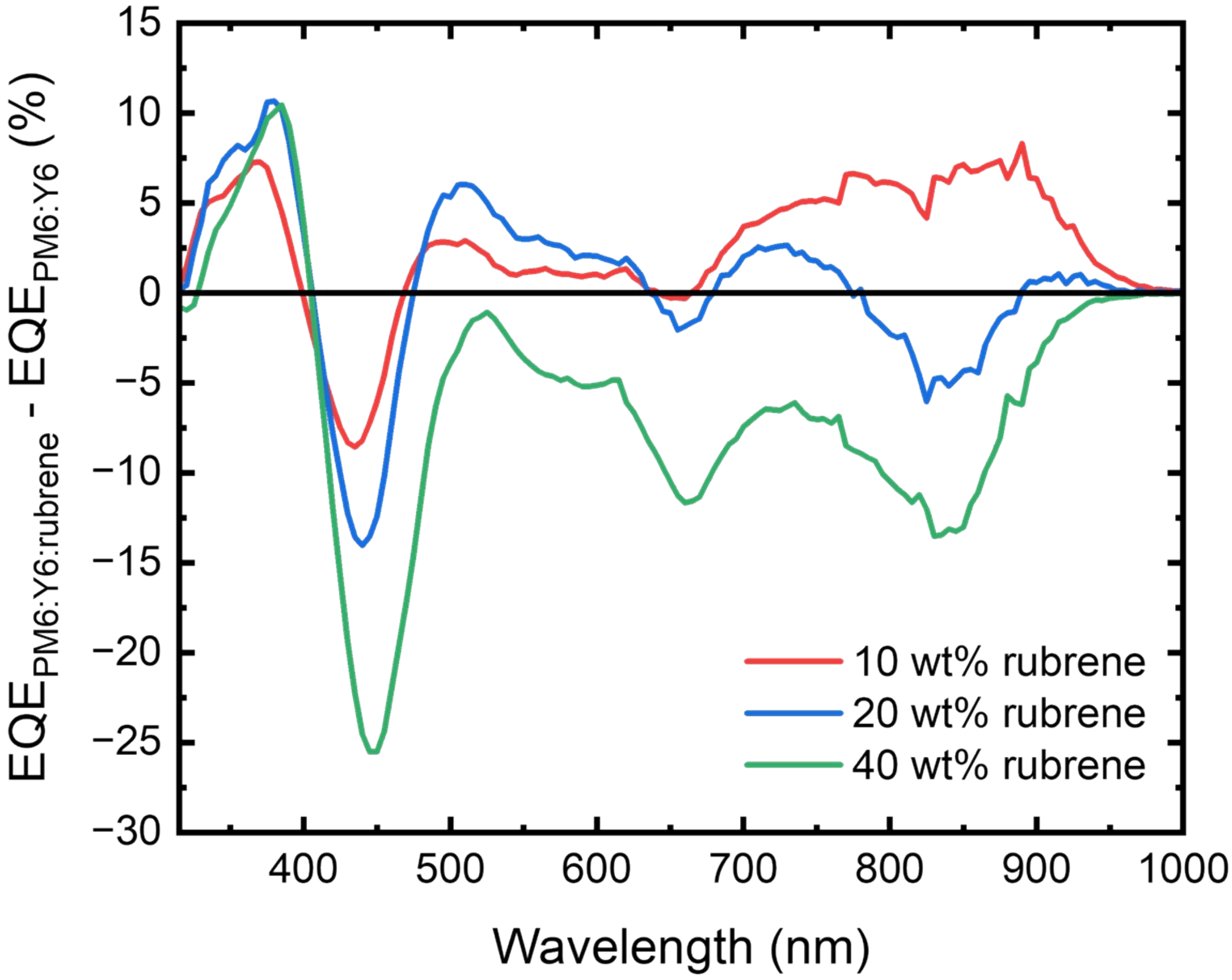

Figure S25. The change in EQE as a function of wavelength between the pristine PM6:Y6 OSC device, and the PM6:Y6 devices with 10, 20, and 40 wt% rubrene doping.

References

1. Luhman, W. A. & Holmes, R. J. Investigation of Energy Transfer in Organic Photovoltaic Cells and Impact on Exciton Diffusion Length Measurements. *Adv. Funct. Mater.* **21**, 764–771 (2011).

2. Koeppe, R. & Sariciftci, N. S. Photoinduced charge and energy transfer involving fullerene derivatives. *Photochem. Photobiol. Sci.* **5**, 1122–1131 (2006).

3. VanDerMeer, B. W. Kappaphobia is the elephant in the fret room. *Methods Appl. Fluoresc.* **8**, 030401 (2020).

4. Benatto, L. *et al.* FRET–Calc: A free software and web server for Förster Resonance Energy Transfer Calculation. *Comput. Phys. Commun.* **287**, 108715 (2023).

5. Karuthedath, S. *et al.* Intrinsic efficiency limits in low-bandgap non-fullerene acceptor organic solar cells. *Nat. Mater.* **20**, 378–384 (2021).

6. Menke, S. M., Luhman, W. A. & Holmes, R. J. Tailored exciton diffusion in organic photovoltaic cells for enhanced power conversion efficiency. *Nat. Mater.* **12**, 152–157 (2013).

7. Zhang, Y. *et al.* Fluorination vs. chlorination: a case study on high performance organic photovoltaic materials. *Sci. China Chem.* **61**, 1328–1337 (2018).

8. Lewitzka, F. & Löhmannsröben, H.-G. Investigation of Triplet Tetracene and Triplet Rubrene in Solution. *Zeitschrift für Phys. Chemie* **150**, 69–86 (1986).

9. Strickler, S. J. & Berg, R. A. Relationship between Absorption Intensity and Fluorescence Lifetime of Molecules. *J. Chem. Phys.* **37**, 814–822 (1962).

10. Izawa, S. & Hiramoto, M. Efficient solid-state photon upconversion enabled by triplet formation at an organic semiconductor interface. *Nat. Photonics* **15**, 895–900 (2021).

11. Yuan, J. *et al.* Single-Junction Organic Solar Cell with over 15% Efficiency Using Fused-Ring Acceptor with Electron-Deficient Core. *Joule* **3**, 1140–1151 (2019).

12. Giannini, S. *et al.* On the role of charge transfer excitations in non-fullerene acceptors for organic photovoltaics. *Mater. Today* **80**, 308–326 (2024).

13. Wang, R. *et al.* Charge Separation from an Intra-Moiety Intermediate State in the High-Performance PM6:Y6 Organic Photovoltaic Blend. *J. Am. Chem. Soc.* **142**, 12751–12759 (2020).

14. Li, J. *et al.* Charge Generation Dynamics in Organic Photovoltaic Blends under One-Sun-Equivalent Illumination Detected by Highly Sensitive Terahertz Spectroscopy. *J. Am. Chem. Soc.* **146**, 20312–20322 (2024).

15. Karki, A. *et al.* The role of bulk and interfacial morphology in charge generation, recombination, and extraction in non-fullerene acceptor organic solar cells. *Energy Environ. Sci.* **13**, 3679–3692 (2020).

16. Wu, T., Ni, W., Gurzadyan, G. G. & Sun, L. Singlet fission from upper excited singlet states and polaron formation in rubrene film. *RSC Adv.* **11**, 4639–4645 (2021).

17. Ma, L. *et al.* Singlet fission in rubrene single crystal: direct observation by femtosecond pump–probe spectroscopy. *Phys. Chem. Chem. Phys.* **14**, 8307 (2012).

18. DuBose, J. T., Szabó, G., Chakkamalayath, J. & Kamat, P. V. Excited-State Transient

Chemistry of Rubrene: A Whole Story. *J. Phys. Chem. A* **126**, 7147–7158 (2022).

19. Zhu, L. *et al.* Efficient Organic Solar Cell with 16.88\% Efficiency Enabled by Refined Acceptor Crystallization and Morphology with Improved Charge Transfer and Transport Properties. *Adv. Energy Mater.* **10**, 1904234 (2020).
